# Delineating species along shifting shorelines: *Tropheus* (Teleostei, Cichlidae) from the southern subbasin of Lake Tanganyika

**DOI:** 10.1186/s12983-018-0287-4

**Published:** 2018-11-13

**Authors:** Maarten Van Steenberge, Joost André Maria Raeymaekers, Pascal István Hablützel, Maarten Pieterjan Maria Vanhove, Stephan Koblmüller, Jos Snoeks

**Affiliations:** 1 Vertebrates Section, Royal Museum for Central Africa, Leuvensesteenweg 13, 3080 Tervuren, Belgium; 2 Laboratory of Biodiversity and Evolutionary Genomics, University of Leuven, Charles Deberiotstraat 32, 3000 Leuven, Belgium; 3 Operational Directorate Taxonomy and Phylogeny, Royal Belgian Institute for Natural Sciences, Vautierstraat 29, 1000 Brussels, Belgium; 4 Institute of Biology, University of Graz, Universitätsplatz 2, 8010 Graz, Austria; 5 Faculty of Bioscience and Aquaculture, Nord University, Universitetsalléen 11, 8026 Bodø, Norway; 6 Flanders Marine Institute (VLIZ), Wandelaarkaai 7, 8400 Oostende, Belgium; 7 Department of Botany and Zoology, Faculty of Science, Masaryk University, Kotlářská 2, 611 37 Brno, Czech Republic; 8 Centre for Environmental Sciences, Research Group Zoology: Biodiversity & Toxicology, Hasselt University, Agoralaan Gebouw D, B-3590 Diepenbeek, Belgium; 9 Zoology Unit, Finnish Museum of Natural History, University of Helsinki, P.O.Box 17, FI-00014 Helsinki, Finland

**Keywords:** Clinal variation, Morphology, Body shape, Meristics, Species delimitation, Africa, AFLP, P_ST_, Population differentiation, Evolution

## Abstract

**Background:**

Species delineation is particularly challenging in taxa with substantial intra-specific variation. In systematic studies of fishes, meristics and linear measurements that describe shape are often used to delineate species. Yet, little is known about the taxonomic value of these two types of morphological characteristics. Here, we used *Tropheus* (Teleostei, Cichlidae) from the southern subbasin of Lake Tanganyika to test which of these types of characters best matched genetic lineages that could represent species in this group of stenotypic rock-dwelling cichlids. We further investigated intra-population variation in morphology. By linking this to a proxy of a population’s age, we could assess the evolutionary stability of different kinds of morphological markers.

**Results:**

Morphological data was collected from 570 specimens originating from 86 localities. An AFLP approach revealed the presence of five lineages in the southern subbasin: *T. moorii*, *T. brichardi*, *T.* sp. ‘maculatus’, *T.* sp. ‘Mpimbwe’ and *T*. sp. ‘red’, which we consider to represent distinct species. Although both types of morphological data supported this classification, a comparison of P_ST_-values that describe inter-population morphological differentiation, revealed a better correspondence between the taxon delineation based on AFLP data and the patterns revealed by an analysis of meristics than between the AFLP-based taxon delineation and the patterns revealed by an analysis of shape. However, classifying southern populations of *Tropheus* was inherently difficult as they contained a large amount of clinal variation, both in genetic and in morphological data, and both within and among species. A scenario is put forward to explain the current-day distribution of the species and colour varieties and the observed clinal variation across the subbasin’s shoreline. Additionally, we observed that variation in shape was larger in populations from shallow shores whereas populations from steep shores were more variable in meristics. This difference is explained in terms of the different timescales at which small and large scale lake level fluctuations affected populations of littoral cichlids at steep and shallow shores.

**Conclusions:**

Our results showed meristics to be more evolutionary stable, and of higher taxonomic value for species delimitation in *Tropheus*, than linear measurements that describe shape. These results should be taken into account when interpreting morphological differences between populations of highly stenotypic species, such as littoral cichlids from the Great East African Lakes.

**Electronic supplementary material:**

The online version of this article (10.1186/s12983-018-0287-4) contains supplementary material, which is available to authorized users.

## Background

Although species represent fundamental entities in biology, the definition of a species and the criteria that a group of organisms should fulfil to be considered a species remain a source of debate amongst systematists [1, 2]. Most notably, recently-evolved species, or species that are part of a radiation, are difficult to delineate by either morphological or molecular tools [3]. A major difficulty in the delineation of such species lies in the occurrence of both small inter- and large intra-specific differentiation, which can even be part of a continuum. This is especially the case for many cichlids from the East African Great Lakes [4–6], such as the Lake Tanganyika endemic *Tropheus* Boulenger, 1898. This genus of highly stenotypic algae scrapers from the lake’s rocky shores harbours substantial variation in molecular [7], chromatic [8, 9], and morphometric traits [10, 11], or in a combination of the above [12, 13]. Using *Tropheus* as a model, we will provide guidelines on how to distinguish intra- from interspecific variation in cichlid radiations.

For fishes, species delineation is traditionally based on morphological traits that include linear measurements (shape), counts of scales, fin rays, teeth, and gill rakers (meristics), or colour patterns [6, 14]. Little attention has, however, been given to the interpretation of these traits. As with molecular markers [15], different morphological characters can reveal different and even conflicting patterns in systematic studies of fishes [16]. As speciation is inherently a complex and multi-layered process that, in Great Lake cichlids, is often still ongoing [17], such conflicting patterns can even be expected. This calls for an integrative approach in species delineation taking the ecology, behaviour, parasitology, evolutionary history and population structure of the taxon into account [18, 19]. For geographically structured taxa, this would also require the study of a large number of specimens from many sites. However, as hundreds of Great Lake cichlid species still await formal description [20], and as many of these species are elusive, acquiring such information for all of them would be, if even possible, a Herculean endeavour. However, thanks to large museum collections, and its long-standing use as a model taxon for studies in behavioural ecology and speciation, such an approach can be adopted for *Tropheus*. By contrasting patterns obtained from different types of morphological characters with our knowledge of the genus’ evolution, behaviour and ecology, we will be able to interpret the systematic value of the different morphological traits.

*Tropheus* are highly specialised littoral algae scrapers that are generally restricted to the upper 20 m (30 m for the basal species *T. duboisi*) of Lake Tanganyika’s rocky shores. They are sexually monochromatic, maternal mouthbrooders and highly stenotypic [8, 9]. The genus contains over a hundred, mostly geographically isolated, colour varieties. Genetic drift, sexual and social selection, parasite-mediated selection and isolation by distance can all be invoked to explain some of the differences observed between *Tropheus* populations [21–24]. Yet, these mechanisms fail to explain the more complex distribution patterns observed today, both in terms of species as in terms of colour varieties and genetic lineages [13, 25]. Often, the distributions of littoral Lake Tanganyika cichlids can be explained by taking climate-driven fluctuations in lake level into account [26]. The most dramatic of these events probably divided the lake into three, possibly four, isolated paleolakes that correspond with the present day subbasins [27]. As these subbasins still differ in the composition of their cichlid communities [28], these ancient events remain the baseline to explain distribution patterns in Lake Tanganyika cichlids today [5].

Also on a smaller scale, and without subdividing the lake, drops and rises in water level profoundly influenced the evolutionary history of littoral cichlids. As every such event displaced the shoreline, littoral cichlids, including *Tropheus*, were forced to colonise new areas. Therefore, periodic lake level changes resulted in a dynamic pattern of local extinctions, recolonisations, population splitting and admixture [13, 29–32]. Especially in shallow parts of the lake, where even a relatively minor drop in water level resulted in a large displacement of the shoreline, such events profoundly influenced the population history of littoral cichlids [29, 32–35]. Although the effects of lake level changes on the genetic structure of some *Tropheus* populations are well documented, their effects on the morphological differentiation in the genus remain largely unknown.

We studied morphological differentiation in *Tropheus* populations from the southern subbasin of Lake Tanganyika. Although species of *Tropheus* occur throughout the lake, we chose to focus on southern populations for a variety of reasons. Firstly, the boundaries of the southern basin can be rather easily defined and its geomorphology is relatively well known. Secondly, whereas instances of sympatry between *Tropheus* species are not uncommon in parts of the northern and especially the central subbasin, this situation is rare in the south. Hence, whereas sympatric species of *Tropheus* in the other subbasins differ in habitat preference [11], allopatric *Tropheus* populations in the southern subbasin are ecologically highly similar. This is important when studying shape, which was shown to be influenced by ecology in *Tropheus* [10]. Thirdly, southern colour varieties of *Tropheus* are much better known than those of the central and northern subbasins and they are also better represented in phylogenetic studies [36]. Hence, we know that *Tropheus* in the southern subbasin contains several well-separated genetic lineages, most likely corresponding to species, but also some varieties that resulted from inter-specific hybridisation [13, 37, 38]. Finally, whereas large areas of shoreline of the northern and the central subbasin remained largely unexplored, historical and recent collection efforts resulted in relatively large collections originating from many locations along the southern shores [5, 28, 39].

The aim of this study is twofold. At first, we want to investigate which morphological traits, traditionally used in ichthyology, are most suited for species delineation in cichlid radiations. Secondly, we will compare the rate of morphological differentiation in measurements and meristics. For this, we will investigate the morphological variation within and between populations of *Tropheus*. For the first aim, we will contrast genomic differentiation obtained from AFLP scans, a method proven to be suitable for species delineation in *Tropheus* [13, 38], with morphological differentiation in measurements and meristics. This will tell us to what extent inter-specific differentiation exceeds intra-specific variation for both types of morphological data. For the second aim, we will compare the variation in meristics and shape within populations of *Tropheus*. This will be examined in relation to the bathymetry of the shoreline at which the population occurs. Here, the slope of the shoreline, steep or shallow, is used as a proxy for the stability, and age, of its *Tropheus* population. As *Tropheus* is restricted to the upper layers of the shoreline, populations from steep shores will mostly be displaced vertically when the lake level changes. At shallow shores, lake level changes will, in addition, cause large horizontal displacements of the shoreline. Additionally, the suitable habitat for *Tropheus* is much more patchy at shallow than at steep shores. Due to higher sedimentation rates, rocky outcrops along shallow shores can be considered islands in a sea of sand. At steep shores, however, large zones of suitable habitats are available for rock-dwelling cichlids. Hence, due to large-scale lake level fluctuations, shallow shores provide less stable habitats for populations of *Tropheus*, which will be much more affected by admixture or extinction events. Hence, populations of *Tropheus* along shallow shores can be considered less stable and younger, whereas those from steep shores, which had a more stable demographic history, can be considered older. Previously, Nevado et al. [31] found that populations of *Tropheus* from shallow shores were more diverse in mitochondrial markers than those from steeper shores. This was interpreted as being caused by more frequent events of population admixture. We hypothesise that a similar pattern is also present in morphological characteristics and that populations from the shallow shores are more variable in morphology than populations found at the steeper parts of the lake. Next to shedding new light on the effects of climate-driven population dynamics on the morphological evolution of littoral cichlids, this will also allow us to interpret both the morphological variation present within a species and between species. This will help to define criteria for species delineation in Great Lake cichlids using morphological data.

## Methods

**Fig. 1:**
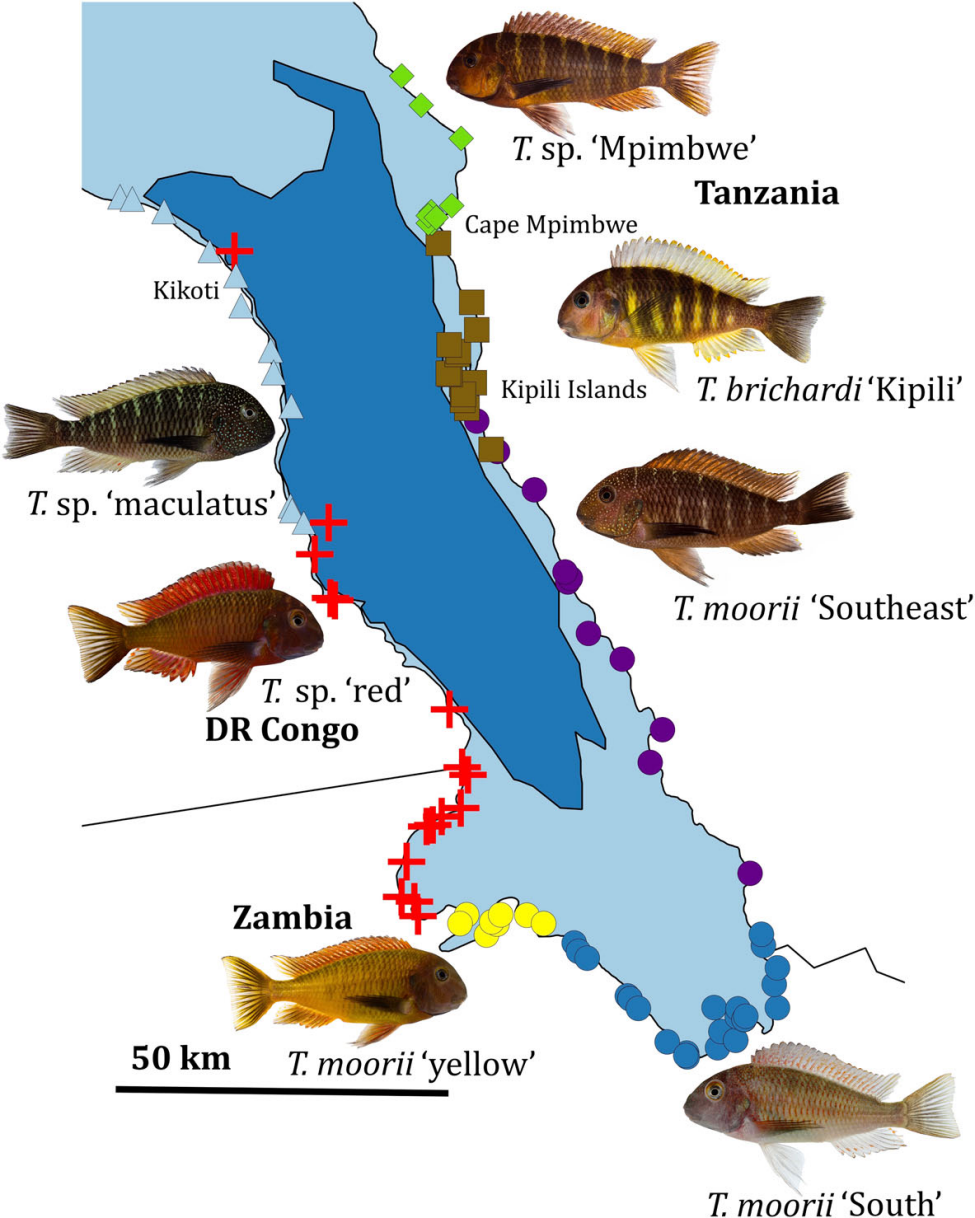
Map of Lake Tanganyika**.** Indicating the 94 collection localities of the 570 specimens of *Tropheus* studied for morphology and 108 specimens studied genetically. The dark blue area denotes the zone deeper than 600 m below the current lake level, corresponding to the southern paleolake

In total, 108 specimens of *Tropheus* were included in the molecular analysis, while 570 specimens were examined morphologically. Specimens used for the morphological analysis stem from several historical and recent collections. As these were often no longer useful for molecular analyses, AFLP fingerprints were often taken from specimens obtained from the aquarium trade. As the origin of *Tropheus* populations can be readily identified based on their colour patterns, and as we only worked with reliable suppliers, we can to a high degree be confident about their origin. Yet, given possible aquarium effects on morphology, these specimens were not used for the morphological study. Hence, many specimens were not used for both approaches. Additionally, due to differences in logistical feasibility and political stability in the study region, a completely balanced sampling could not be achieved (Fig. 1, Additional file 1). Specimens were classified into five species and seven groups based on aquarists’ classifications [9, 40] and previous studies [13, 38]. These are, listed by their distribution from the northwestern- to the northeasternmost part of the subbasin: *T.* sp. ‘maculatus’, *T*. sp. ‘red’, *T. moorii* ‘yellow’, *T. moorii* ‘South’, *T. moorii* ‘Southeast’, *T. brichardi* ‘Kipili’, and *T.* sp. ‘Mpimbwe’. Three of these groups: *T. moorii* ‘yellow’, *T. moorii* ‘South’ and *T. moorii* ‘Southeast’, will be referred to as *T. moorii* s.l. (sensu lato) collectively, even though previous studies demonstrated that *T. moorii* ‘yellow’ is a natural hybrid between *T. moorii* ‘South’ and *T.* sp. ‘red’ [13, 38, 41]. Whereas morphological and molecular data supported this classification, providing formal descriptions of these species falls out of the scope of this study as this would require a comparison with type specimens of species of *Tropheus* described from the northern and central subbasins. Specimens collected at different locations will be treated as belonging to different populations. At two sites along the western, Congolese, shore - Kikoti and Kasenga (not to be confused with Kasenga, Zambia) - representatives of more than one group were found.

For 108 specimens of *Tropheus*, collected at 34 localities in the southern subbasin of Lake Tanganyika, AFLP genotyping was performed (Fig. 1, Additional file 1). DNA was extracted from ethanol-preserved finclips using proteinase *K* digestion and protein precipitation with ammonium acetate. AFLP genotyping followed the protocol described in [38]. Ten primer combinations were used for selective amplification: EcoRI-ACA/MseI-CAA, EcoRI-ACT/MseI-CAG, EcoRI-ACC/MseI-CAC, EcoRI-ACA/MseI-CAG, EcoRI-ACA/MseI-CAC, EcoRI-ACA/MseI-CAT, EcoRI-ACT/MseI-CAT, EcoRI-ACT/MseI-CAA, EcoRI-ACT/MseI-CAC, EcoRI-ACC/MseI-CAA. Selective PCR products were sized against an internal standard (GeneScan-500 ROX, Applied Biosystems) on an ABI 3130xl automated sequencer (Applied Biosystems). The AFLP data are part of a larger dataset (Van Steenberge, unpublished data) of 208 AFLP *Tropheus* fingerprints in which negative controls and a minimum of 19 replicates per primer combination were included in the reactions. The data included 44 newly generated fingerprints, but also AFLP fingerprint data of 70 samples obtained from [41]. The latter were generated in the same facilities and under the same conditions and compatibility of the data was guaranteed by including six samples in both datasets and by analysing them as replicates. Size and peak height of fragments between 100 and 500 bp were determined using GeneMapper v.3.7 (Applied Biosystems). Bins were checked by eye and pre-processed for threshold optimization for locus retention and phenotype calling with AFLP-SCORE 1.4a [42] following [41]. The average mismatch error rate was 1.26%. The final binary matrix for the representative set of southern *Tropheus* consisted of 248 polymorphic characters.

Genetic clusters were identified by performing a principal component analysis (PCA) on the correlation matrix of the presence-absence matrix of AFLP data. Additionally, non-metric multidimensional scaling (NMDS) was performed, using Jaccard’s similarity index. Clinal variation in AFLP data was examined by plotting values for the main principal components (PC) versus the geographic distance measured along the shoreline, starting from the northwesternmost locality. Clinal variation was visually inspected by performing LOESS smoothing [43] with a fixed smoothing parameter of 0.5. Clinal variation in genetic similarity was also examined without dimension reduction. For this, Mahalanobis distances were calculated between AFLP fingerprints, and the Pearson correlation matrix of these distances was visualised by means of a heat map. HExT [44] was used to infer a Neighbour Joining (NJ) tree based on Nei-Li distances [45]. This tree was rooted with three individuals of *T. duboisi*, which represents the sister group of all other *Tropheus* species [46]. Statistical support was estimated by performing 1000 bootstrap replicates. Finally, Structure v2.3.4 [47] was run with 20 replicates (400,000 generations following a burn-in of 200,000 generation) for K ranging from 1 to 10, assuming the admixture model and correlated allele frequencies. The most likely number of clusters was determined in Structure based on the ΔK statistics [48]. However, given our unbalanced sampling of populations, this method might not be appropriate to find the true number of groupings in the dataset. Hence, solutions for other values of K were also examined.

A total of 570 specimens originating from 86 locations along the shoreline of the southern subbasin of Lake Tanganyika were used in the morphological analysis (Fig. 1, Additional file 1). Most specimens originated from the collections of the Royal Museum for Central Africa (RMCA), but specimens from the Natural History Museum (BMNH), the Muséum National d’Histoire Naturelle (MNHN), the Royal Belgian Institute of Natural Sciences (RBINS) and from the private collection of Heinz Büscher (University of Basel) were also included. For the nominal species that have their type locality in the southern subbasin, the complete type series was studied with the exception of non-alcohol-preserved specimens (skeletons). For each specimen, 23 measurements and 16 meristics were taken, following [12] (Additional file 1).

Principal component analyses were performed on the correlation matrix of the meristics and on the covariance matrix of the log_10_-transformed measurements. This is standard practice in ichthyology, as meristics were taken from widely distinct structures, whereas measurements were all expressed in mm [6]. Meristics that could not be obtained were treated as missing data. As the number of caudal peduncle scales was always 16, this variable was excluded from the analyses. Morphology was studied on the three main PC's that describe variation in meristics and shape, respectively. The first PC of the log-transformed measurements described generalised size [49]. Therefore, only the second, third and fourth PC were used as the components describing shape. Linear regressions with standard length (SL) confirmed that these three axes were unrelated with size (*p* < 0.05). As some meristics can change with size (e.g. teeth counts, gill rakers), the effects of size were removed from the main PC’s of the meristics. For this, linear regression with SL was performed and residuals, labelled size-reduced PC (SR-PC), were studied instead.

Clinal variation in morphology was investigated by plotting values for the main components that explain variation in meristics and shape versus geographical distances taken along the shoreline, starting from the northwesternmost locality. Clinal patterns and sharp differentiation between neighbouring populations (localities), were visually investigated on these plots by performing LOESS smoothing [43] with a fixed smoothing parameter of 0.5. To investigate whether clinal patterns were also visible on the original variables, this analysis was repeated on each of the meristics and measurements separately. As the patterns proved to be less pronounced, a fixed smoothing parameter of 0.2 was used instead. For this analysis, measurements taken on the head were expressed as percentages of head length, and those taken on the rest of the body as percentages of standard length. Morphological inter-population differentiation between all 67 populations (i.e. sampling sites) that contain more than two individuals was investigated by means of P_ST_. P_ST_ quantifies the proportion of among-population phenotypic variance in quantitative traits, and was calculated following [50]. Inter- and intra-specific differentiation was compared using non-parametric Kruskall-Wallis tests, complemented with Dunn’s post-hoc tests. As some lineages of *Tropheus* were known to be of hybrid origin, a distinction was made between “true” inter- or intra-specific differentiation and “distant” intra-specific differentiation. The latter category included comparisons between populations of hybrids and their presumed parent taxa and between conspecific populations that differed profoundly in their AFLP signature (see below). Hence, comparisons between *T. moorii* ‘yellow’ (loc. 27–33) and southern *T.* sp. ‘red’ (loc. 19–26) and *T. moorii* ‘South’ (loc. 34–59), and between *T. moorii* ‘Southeast’ (loc. 60–72) and *T. moorii* ‘South’ (loc. 34–59) were treated as “distant” intra-specific.

The relation between the amount of morphological variation within a population and the slope of shoreline at which it occurs was investigated, using the data of 549 specimens from all 67 populations for which two or more specimens could be examined. The different localities were classified as steep or shallow shores, based on their distance to the -600 m bathymetric line, using the bathymetric map presented in [51]. This depth was chosen to model the contours of the southern paleolake, following Nevado et al. [31]. Morphological variation within a population (locality) was quantified by the variances of the three main PC’s explaining variation in shape and by the three main SR-PC’s of the meristics. In order to capture more of the morphological variance, the weighted sum of these three variances, i.e. the sum of the variances of the PC scaled by the percentage of variance explained by this PC, was calculated. Variances of the components and their weighted sums were compared between populations from shorelines with different slopes using Mann-Whitney U tests. Statistical analyses were performed using Past [52] and R [53].

## Results

### AFLP scans

**Fig. 2:**
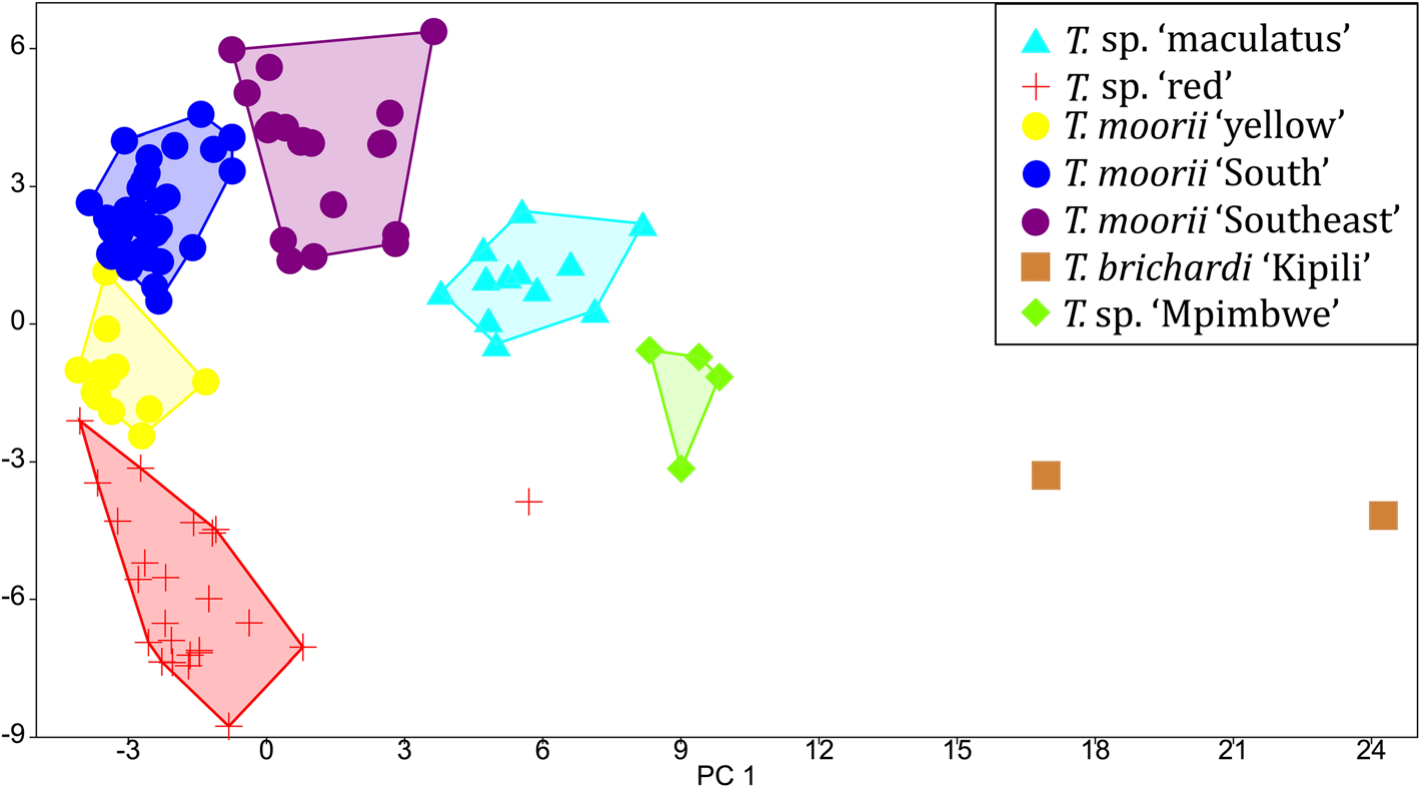
PC2 vs. PC1 of the PCA calculated on 108 AFLP fingerprints. The northernmost specimen of *T.* sp. ‘red’ (Kikoti, loc. 4) is visualised separately as it is not included in the convex hull with the other specimens of *T.* sp. ‘red’, PCA was calculated on the correlation matrix

**Fig. 3:**
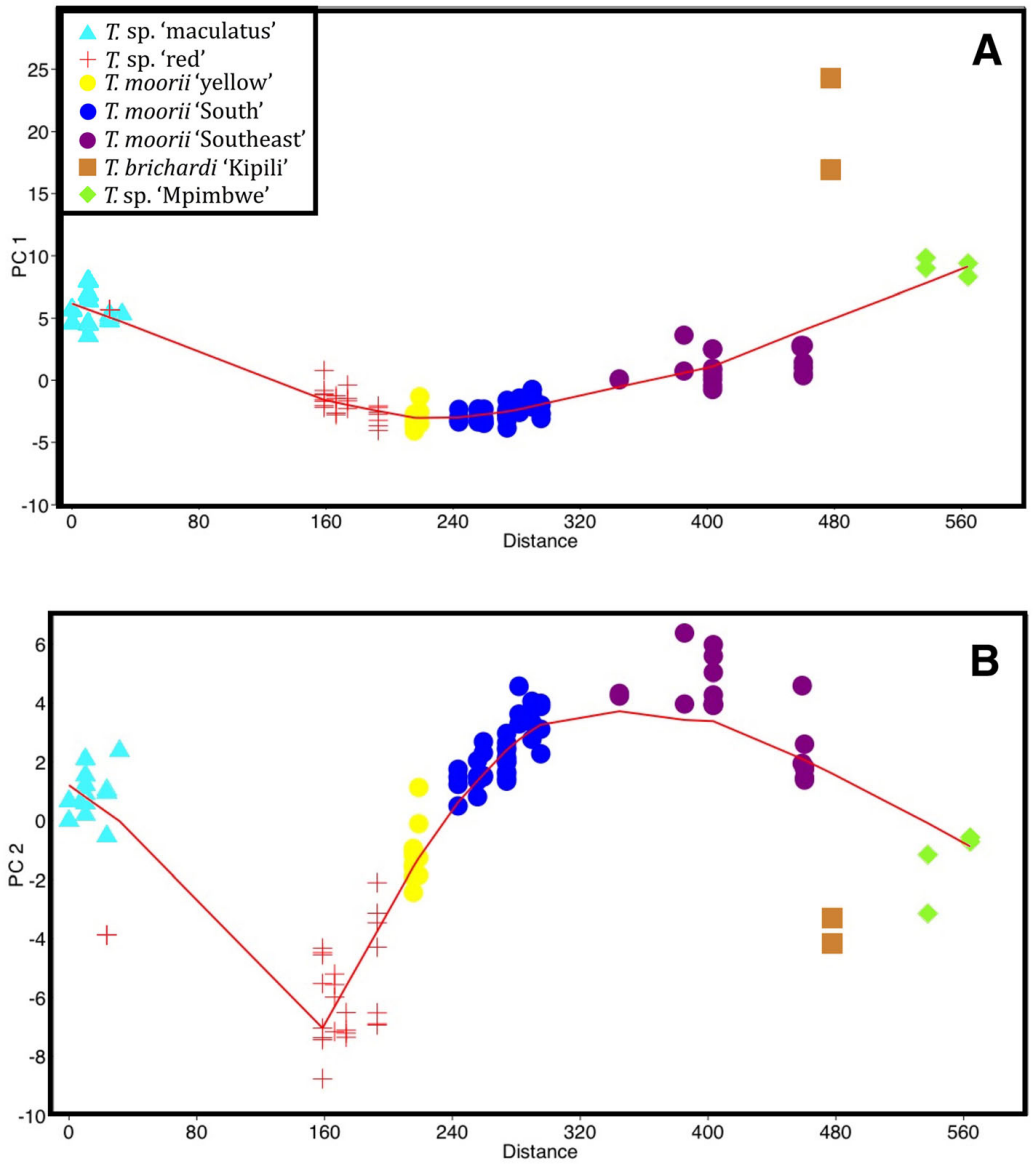
PC1 (**a**) and PC2 (**b**) of the PCA calculated on 108 AFLP fingerprints vs. geographical distance. Distance was taken along the shoreline (in km) starting from the northwesternmost locality, LOESS curves were calculated with a fixed smoothening parameter of 0.5, PCA was calculated on the correlation matrix

A PCA was performed on the correlation matrix of all 108 AFLP fingerprints. Although the two main PC’s only explained 8.2% and 5.6% of the variance, most of the groups could be separated using these axes (Fig. 2). The first PC foremost showed the distinction of *T. brichardi* ‘Kipili’, with high values, from the other groups. Specimens belonging to *T. moorii* s.l. and *T*. sp. ‘red’ had low values for this PC, although values for specimens belonging to *T. moorii* ‘Southeast’ were somewhat higher than those of its conspecifics. Values for this axis were intermediate for specimens belonging to *T*. sp. ‘maculatus’ and *T*. sp. ‘Mpimbwe’. The same held for the sole *T*. sp. ‘red’ specimen collected at Kikoti, which had a higher value for PC1 than its conspecifics that were collected further south. The second PC separated specimens belonging to *T. moorii* ‘South’ and ‘Southeast’ from all other groups, although values for *T. moorii ‘*yellow’, and *T*. sp. ‘maculatus’ were somewhat intermediate. When the two main PC’s were presented versus locality (Fig. 3), clinal patterns emerged. For PC1, clinal variation was observed between all groups, with the exception of *T. brichardi* ‘Kipili’. For PC2, clinal variation was found between *T.* sp. ‘red’ (with the exception of the northernmost specimen from Kikoti), *T. moorii* s.l. and T. sp. ‘Mpimbwe’. Hence, for PC1, *T. brichardi* ‘Kipili’ did not abide to a clinal pattern whereas this held for *T. brichardi* ‘Kipili’ and *T.* sp. ‘maculatus’ for PC2. The NMDS plot was highly similar to the results of the PCA performed on the correlation matrix (Additional file 2).

**Fig. 4:**
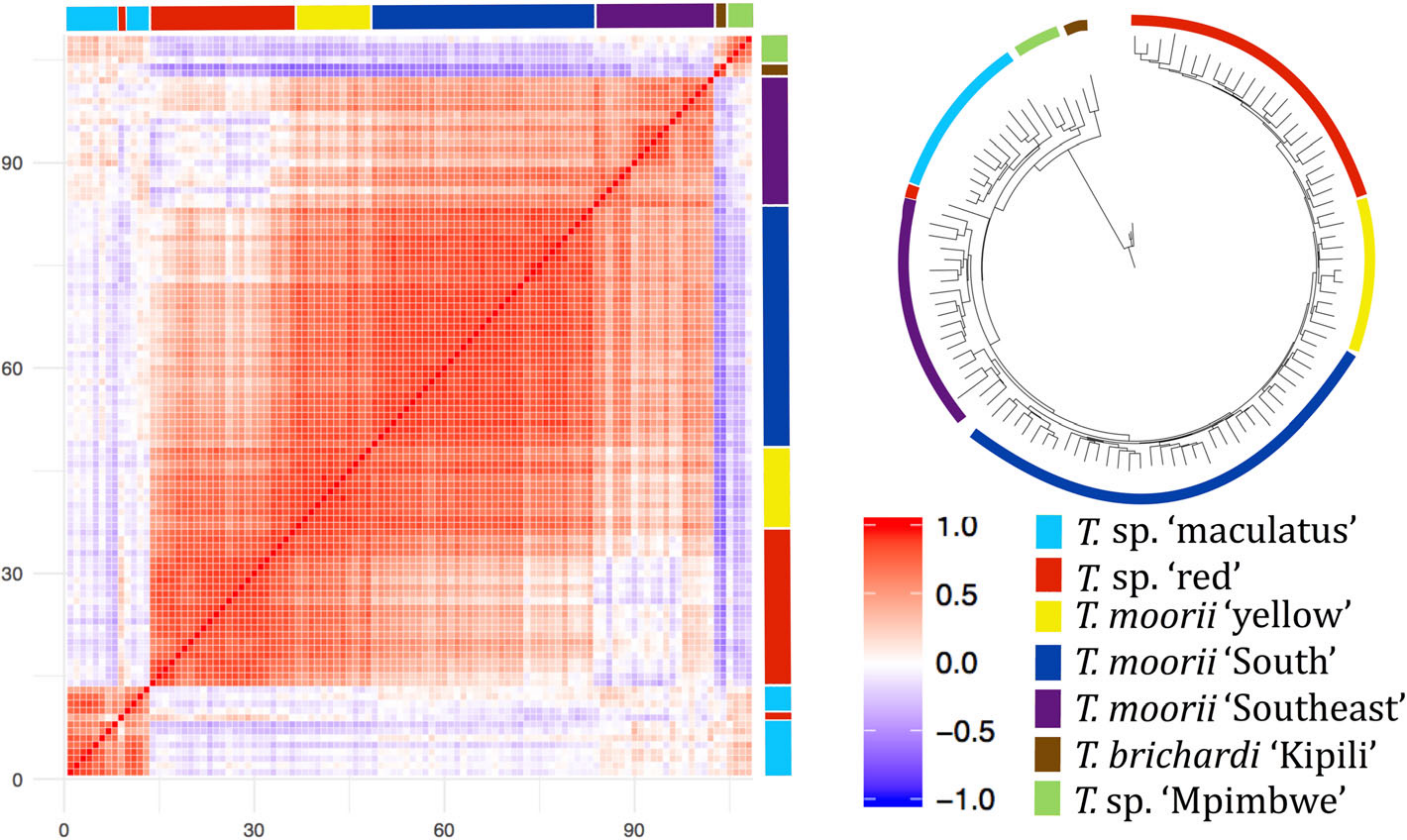
Heat map of genetic similarity and AFLP-based NJ tree of 108 southern specimens of *Tropheus*. Genetic similarity was calculated using Mahalanobis distances between AFLP fingerprints, and visualised on the heat map via the Pearson correlation matrix, using the red-blue colour scale. Specimens are grouped according to their origin along the lakeshore, with specimens from the northwesternmost locality bottom-left. The NJ tree was constructed using three specimens of *T. duboisi* as the outgroup. Colours next to the heat map and around the tree denote group membership. The *T*. sp. ‘red’ specimen that is visualised separately in the NJ tree and in the heatmap is the sole specimen from Kikoti (loc. 4). A more detailed version of the NJ tree is presented in Additional file 3

As the first two PC’s only explained 13.6% of the variance, clinal patterns were also examined on the entire dataset by constructing a heat map of the Pearson correlation matrix of genetic similarity (Fig. 4). This figure again supported the distinction of *T*. sp. ‘maculatus’, *T. brichardi* ‘Kipili’ and *T*. sp. ‘Mpimbwe’ from the other groups. Within the combined *T.* sp. ‘red’ and *T. moorii* s.l. clades, genetic similarity was also high within groups. Between groups, however, a clinal pattern was seen, with specimens of *T.* sp. ‘red’ being highly similar to *T. moorii* ‘yellow’, relatively similar to *T. moorii* ‘South’ and often rather distinct from *T. moorii* ‘Southeast’. This analysis also indicated that the *T*. sp. ‘red’ specimen from Kikoti shared genetic similarity with both all the other specimens of *T.* sp. ‘red’ and with specimens of *T.* sp. ‘maculatus*’*, with which it co-occurs. Finally, the analysis also reveals some similarity between *T.* sp. ‘maculatus*’* and *T.* sp. ‘Mpimbwe’.

A NJ tree was also constructed (Fig. 4, Additional file 3). This showed the distinctness of *T. brichardi* ‘Kipili’, *T*. sp. ‘Mpimbwe’, *T*. sp. ‘maculatus’, and the *T*. sp. ‘red’ specimen from Kikoti, which were –in this order – resolved as sister groups of a larger ‘southern’ clade. Within the latter, two clades of *T. moorii* ‘Southeast’ branched off first, followed by a clade comprised of *T. moorii* ‘South’, *T. moorii* ‘yellow’ and *T.* sp. ‘red’. In this last clade, internal nodes were poorly resolved. When *T. moorii* ‘yellow’, a known hybrid taxon [13], was removed, the branching order of the remaining groups and clades in the resulting NJ tree did not change.

The Bayesian inference of population structure revealed a peak in ∆K [48] for K = 2. The resulting solution supported a separation between *T*. sp. ‘Mpimbwe’, *T. brichardi ‘*Kipili’ and *T*. sp. ‘maculatus’ on the one hand, and *T.* sp. ‘red’, *T. moorii* ‘yellow’ and *T. moorii* ‘South’ on the other (Additional files 4,5). Samples belonging to *T. moorii* ‘Southeast’ were partially assigned to both clusters. The second best supported clustering was for K = 5. Here, the clusters were: i) *T.* sp. ‘maculatus’, ii) *T.* sp. ‘red’, iii) *T. moorii* ‘South’, iv) *T. moorii* ‘Southeast’, and v) *T. brichardi* ‘Kipili’ and *T.* sp. ‘Mpimbwe’. Samples belonging to *T. moorii* ‘yellow’ were intermediate between *T.* sp. ‘red’ and *T. moorii* ‘South’, whereas some of the southernmost samples of *T. moorii* ‘Southeast’ had a high affinity with *T. moorii* ‘South’. Solutions for higher values of K either further supported the groups as indentified above and/or revealed the clinal pattern between *T.* sp. ‘red’ and *T. moorii* ‘South’, with intermediate values for specimens belonging to *T. moorii* ‘yellow’. For higher values of K (K > 5), the distinction between *T. brichardi* ‘Kipili’ and *T.* sp. ‘Mpimbwe’ became supported. Additionally, more complex patterns became present in *T. moorii* ‘Southeast’ (K > 6), which could point at a higher degree of population structure in that group. In general, we noted that our heavily unbalanced sample sizes across the various *Tropheus* taxa, which is in part due to differences in accessibility for sampling in certain areas, might have biased the inference of genetic clusters in Structure.

To conclude, AFLP scans mostly supported the delineation of the seven groups and five species. Support, however, was weaker for *T*. sp. ‘red’, for which specimens from the southern and northern parts of its distribution did not cluster together in the NJ tree or the PCA. It should be noted, however, that no tissue samples were available for most of this species’ distribution range, and that samples included in the AFLP dataset all stem from the northern (Kikoti) or southernmost parts of its range. However, in a multi-gene phylogeny, the sample from Kikoti clustered with specimens from more southern locations (Singh, unpublished data). AFLP scans further also revealed clear clinal patterns, both within and between groups. This was most evident for *T. moorii* ‘yellow’, which was intermediate between *T.* sp. ‘red’ and *T. moorii* ‘South’. Yet, also *T. moorii* ‘Southeast’ had some mixed affinity with both *T. moorii* ‘South’ and with *T.* sp. ‘Mpimbwe’. Finally, both the Loess curves, the heat map and the Structure analyses revealed similarity between groups occurring at opposite shorelines: *T.* sp. ‘maculatus’ and *T.* sp. ‘Mpimbwe’. Hence, these results suggested that all groups and species in the subbasin, except for *T. brichardi* ‘Kipili’, belong to a single southern lineage.

#### Meristics

**Fig. 5:**
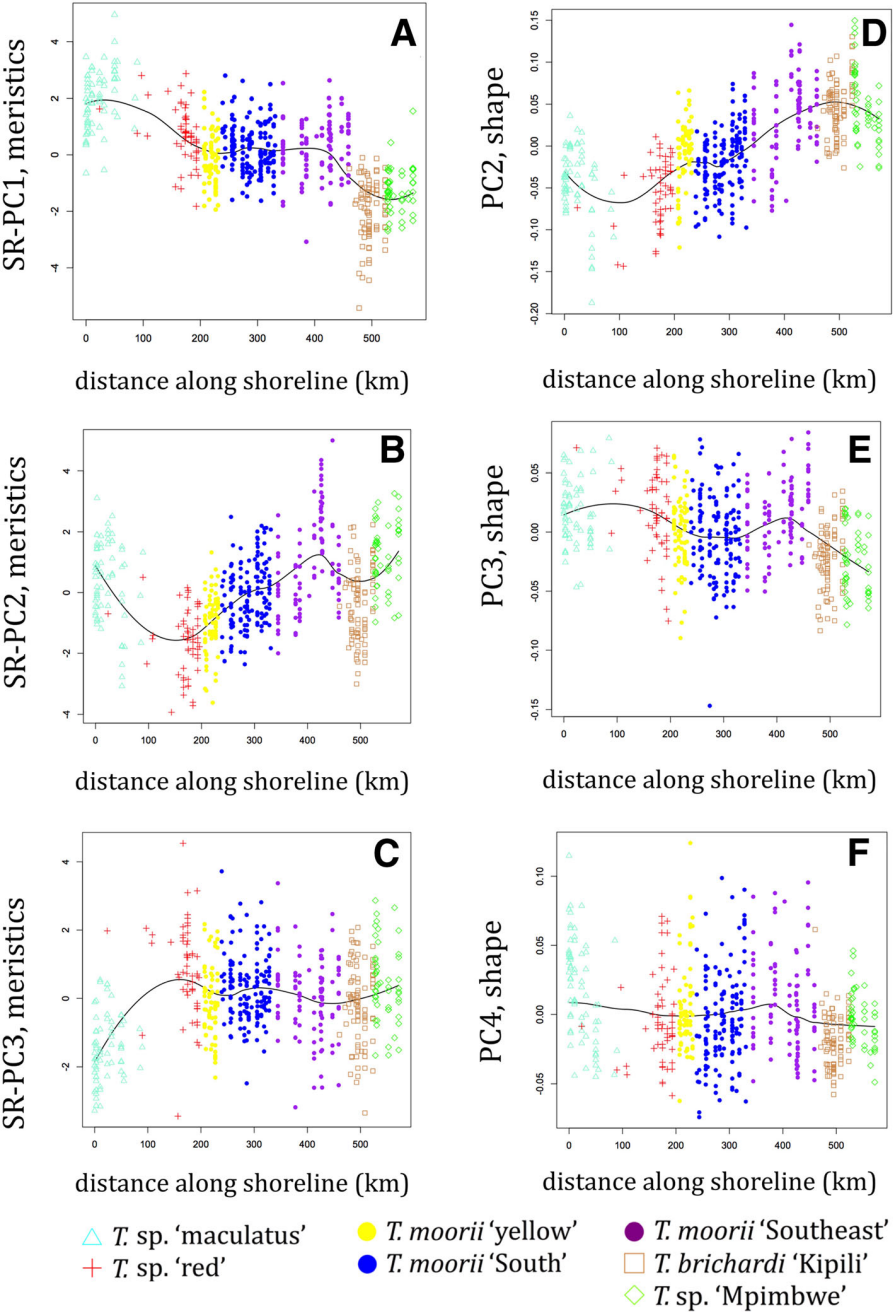
Morphological variation vs. geographical distance along the shoreline. Variation in meristics was descibed by SR-PC1 (**a**), SR-PC2 (**b**), SR-PC3 (**c**) calculated on 15 meristics, variation in shape was described by PC2 (**d**), PC3 (**e**) and PC4 (**f**) calculated on 23 log-transformed measurements for 570 specimens from 86 locations. The distance was taken along the shoreline (in km) starting from the northwesternmost locality. LOESS curves were calculated with a fixed smoothening parameter of 0.5

A PCA was performed on 15 meristics of 570 specimens (Additional file 1). The first PC was dominated by both counts of bicuspid teeth (UBT, LBT) and of anal and dorsal soft rays (ASR, DSR) on the positive side, and by the number of anal and dorsal spines (ASp, DSp) on the negative side. Counts of cheek scales (LChS, TChS) had the highest contribution to PC2 whereas the number of longitudinal line scales (Long) and dorsal and anal spines (DSp, ASp) were the dominant contributors to PC3. As linear regressions with SL revealed that these three PC’s were correlated with size, size-reduced PC’s (SR-PC’s) were investigated. Although a large amount of overlap was seen between the groups, when SR-PC’s were presented versus locality (Fig. 5a-c), a mixed pattern of between-species differentiation and clinal variation within species emerged. The former was observed in all three SR-PC’s, whereas the latter was mostly visible in SR-PC2. For SR-PC1, the LOESS curve contained two clear bending points. These indicated the distinction of *T.* sp. ‘maculatus’, with the highest values, and of *T.* sp. ‘Mpimbwe’ and *T. brichardi* ‘Kipili’, with the lowest, from all other groups. The LOESS curve fitted on SR-PC2 also contained bending points at similar locations although values largely overlapped between *T. moorii* ‘Southeast’ and *T. brichardi* ‘Kipili’ and values for *T.* sp. ‘Mpimbwe’ were higher than those of *T. brichardi* ‘Kipili’. SR-PC2 further showed a clear clinal pattern within the combined groups of *T.* sp. ‘red’ and *T. moorii* s.l. with specimens of *T.* sp. ‘red’ from near the Congo-Zambia border having the lowest, and specimens of some populations of *T. moorii* ‘Southeast’ having the highest values. The third SR-PC, reaffirmed the distinction of *T*. sp. ‘maculatus’ from the other groups. Similar exploratory analyses were performed individually for each of the meristics (Additional file 6). Here, (clinal) differences between groups were less pronounced, which was exemplified by the fact that a smaller smoothing parameter (0.2) had to be used as most of the LOESS curves obtained with a value of 0.5 showed hardly any pattern. Hence, the more informative PCs were used for all further analyses. Nevertheless, the individual plots revealed that the numbers of dorsal and anal fin rays and spines differed between *T*. sp. ‘maculatus’ and all other groups. Higher numbers of bicuspid teeth and gill rakers were observed in *T.* sp. ‘red’. Yet, this might be because specimens in this group were, on average, larger than those from the other groups. Specimens of *T. brichardi* ‘Kipili’ mostly stood out by the number of scales on the cheeks and in the longitudinal line.

#### Shape

A PCA was also performed on the log-transformed measurements (Additional file 1). As the first PC of log-transformed measurements represented size, this variable was not examined. Linear regression confirmed that PC2, PC3 and PC4 were uncorrelated with SL (*p* > 0.05). The main contributors to PC2 were the four measurements of mouth width (LJF, UJF, UJB, LJB), whereas the length of the caudal peduncle (CPL) dominated PC3 and pre-ventral and –pectoral distances (PrV, PrP) PC4. As for the meristics, the PC’s showed a large degree of overlap between the groups. However, when presented versus locality, a mixed pattern of both inter- and intra-specific clinal variation and inter-specific differentiation was revealed (Fig. 5d-f). The former was observed on PC2 and on PC3 for *T.* sp. ‘red’ and *T. moorii* s.l. The LOESS curve showed an upward trend for values of PC2 from *T*. sp. ‘red’ to *T. moorii* ‘Southeast’, when following the coastline in a counter clockwise way. Within the combined *T*. sp. ‘red’ and *T. moorii* s.l. groups, specimens belonging to *T. moorii* ‘South’ had, on average, the lowest values for PC3. Average values for this PC increased in populations of *T*. sp. ‘red’ and *T. moorii* sl. further north along the eastern and western shoreline. The LOESS curve modelled on this axis, however, contained a clear bending point, which showed the distinction of *T. brichardi* ‘Kipili’ and *T.* sp. ‘Mpimbwe’. Except for a minor local maximum for some populations of *T. moorii* ‘Southeast’, no pattern was present in PC4. However, for both PC2 and PC4, *T*. sp. ‘maculatus’ specimens from southern locations (km 50–90) had, on average, lower values than those from northern locations (km 0–31). These two axes showed similar within-group differentiation in *T.* sp. ‘Mpimbwe’. Here, specimens from southern locations (km 528–529) had, on average, higher values than those from northern sites (km 530–572). Similar visualisations were made for ratios of measurements (Additional file 6). As for the meristics, (clinal) differences between groups were less pronounced than in the analysis of PCs. However, some patterns emerged, especially for jaw measurements (LJL, LJF, UJF, UJB, LJB), and for the eye diameter (ED). Yet, as the latter is known to be negatively allometric in fishes, and as the clinal patterns were better captured using PCs, the latter were used for all further analyses.

#### Morphological population parameters

**Fig. 6:**
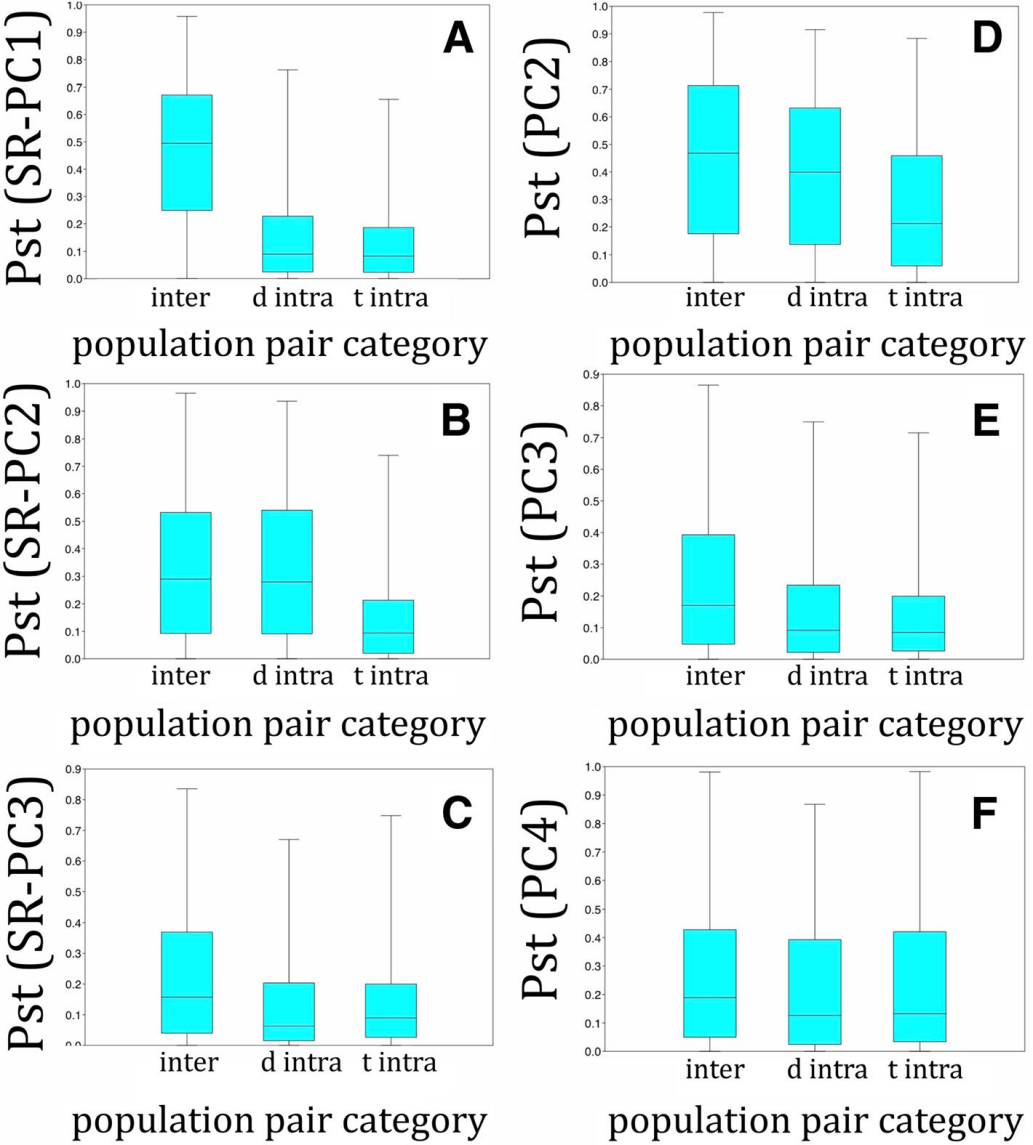
Comparison of intra- and inter-specific morphological differentiation between populations, described by P_ST_ values. Values were calculated on SR-PC1 (**a**), SR-PC2 (**b**), SR-PC3 (**c**) of the 15 meristics and on PC2 (**d**), PC3 (**e**) and PC4 (**f**) of the 23 measurements taken on 549 *Tropheus* specimens from 67 locations. With inter: inter-specific, d intra: distant intra-specific and t intra: true intra-specific comparisons (see text)

Morphological P_ST_ parameters were calculated on each of the three main PCs that describe meristics (SR-PC1–3) and shape (PC2–4). Although our aim was to compare inter- and intra-specific P_ST_-values, this was complicated by the presence of population structure within *T. moorii* s.l. Hence, populations of *T. moorii* s.l. that were partially assigned to *T.* sp. ‘red’ or to *T. brichardi* ‘Kipili’ and *T.* sp. ‘Mpimbwe’ by the Structure analyses and/or by the heat map, were included in the ‘distant’ intra-specific differentiation in the analyses of P_ST_-values. Non-parametric Kruskall-Wallis tests revealed significant differences in morphological population differentiation for each of the six PC’s examined (Table 1, Fig. 6). However, Dunn’s post-hoc tests showed clear differences between meristics and shape. In general, a closer link emerged between species delineation and meristic differentiation than between species delineation and differentiation in shape. For P_ST_-values calculated on meristics, true intra-specific population differentiation was always significantly lower than inter-specific differentiation. For SR-PC1 and SR-PC3, no difference was found between distant and true intra-specific inter-population differentiation, and the former was also significantly lower than inter-specific differentiation. For SR-PC2, however, higher P_ST_ values were recovered for cases of distant intra-specific differentiation than for true intra-specific differentiation, and no difference was found between distant intra-specific and inter-specific differentiation. Different patterns were observed when examining P_ST_-values describing differentiation in shape. Foremost, boxplots (Fig. 6) revealed a much larger overlap between inter- and intra-specific differentiation in shape than in meristics. Additionally, although true intra-specific morphological population differentiation was still significantly lower than inter-specific differentiation for PC2 and PC3, this pattern no longer held for PC4. Even on the main axis describing shape, PC2, the differentiation between *T. moorii* ‘Southeast’ and *T. moorii* ‘South’ was that high that the P_ST_ values calculated between them were significantly larger than both the inter-specific and the other intra-specific comparisons (not shown separately).

**Table 1:** Comparison of inter-population differentiation using P_ST_ values calculated on the three main components that explain variation in meristics (mer, SR-PC1, SR-PC2, SR-PC3) and shape (meas, PC2, PC3, PC4)

mer	Kruskall-Wallis	Dunn		
SR-PC1			dis intra	intra
p	**9.44E-147**	inter	**1.34E-91**	**6.54E-90**
χ^2^	672.5	dis intra		1.07E-01
SR-PC2			dis intra	intra
p	**1.35E-34**	inter	8.44E-01	**5.31E-34**
χ^2^	156	dis intra		**4.16E-25**
SR-PC3			dis intra	intra
p	**3.20E-19**	inter	**5.04E-17**	**2.74E-08**
χ^2^	85.17	dis intra		1.01E-01
meas	Kruskall-Wallis	Dunn		
PC2			dis intra	intra
p	**3.07E-22**	inter	**6.03E-04**	**6.15E-23**
χ^2^	99.07	dis intra		**1.22E-08**
PC3			dis intra	intra
p	**8.48E-18**	inter	**3.79E-11**	**6.74E-13**
χ^2^	78.62	dis intra		2.90E-01
PC4			dis intra	intra
p	**3.81E-04**	inter	**1.38E-04**	5.85E-02
χ^2^	15.75	dis intra		2.01E-01

Results for Kruskall Wallis tests and Dunn’s post hoc test, with: inter: inter-specific, dist intra: ‘distant intra-specific’, and intra: true intra-specific comparisons between populations. Values in bold are significance at the 0.01 level, after sequential Bonferroni correction for 6 (K-W) and 18 (Dunn) comparisons respectively

To conclude, a comparison of inter-population differentiation using P_ST_-values calculated on meristics supported the five species and seven genetic units as defined by the AFLP data. Although significant support for this classification was also obtained using data that describe shape, these patterns were much less clear and the overlap between inter- and intra-specific inter-population differentiation was much higher.

#### Morphological variation vs. shoreline

The variances of the three main axes describing variation in meristics (SR-PC1–3) and shape (PC2–4), as well as their weighted sums, were compared between populations occurring along steep and shallow shores. For the three main SR-PC’s of the meristics, the median variances were higher in populations from steep shores than in populations from shallower parts of the lake. This difference was, however, only significant for SR-PC1 and for the weighted sum of the variances. A different pattern was observed for the data describing shape. Here, specimens from shallow shores had higher mean variances for the three main axes. This was, however, only significant when comparing the weighted sums (Table 2).

**Table 2:** Link between intra-population variation in morphology and the type of shoreline where a population occurs

		shallow	steep	p (Mann-Whitney)
meristics	SR-PC1	0.54	0.71	**0.0345**
	SR-PC2	0.91	1.07	0.3885
	SR-PC3	0.79	1.11	0.1916
	Weighted sum	0.84	1.01	**0.0458**
shape	PC2	6.31E-04	5.71E-04	0.2928
	PC3	7.60E-04	5.14E-04	0.0753
	PC4	5.05E-04	3.11E-04	0.1482
	Weighted sum	7.80E-04	5.96E-04	**0.0417**

Median values of the variances of the six main axes for 29 populations (237 specimens) from shallow and for 38 populations (312 specimens) from steep shores are presented for the six main axes describing variation in meristics and shape. Values in bold are significant at the 0.05 level in non-parametric Mann-Whitney U tests

## Discussion

### Colonisation history of the southern subbasin

Representatives of *Tropheus* from the southern subbasin of Lake Tanganyika exhibit both clear inter- and intra-specific morphological variation, but also a broad spectrum of differences that are less trivial to categorise. The clearest inter-specific differences were found between *T. brichardi* ‘Kipili’ and the other groups, all of which belong to a single southern lineage that is near-endemic to the subbasin [38]. Clear intra-specific differences were observed in *T. moorii* s.l., in which variation of all data types followed a clinal pattern. In many cases, however, our data revealed a complex mixture of bending points and gradual clines that were less straightforward to interpret. As these patterns were shaped by the genus’ complex evolutionary history in the subbasin, their interpretation necessitates a hypothesis regarding these past events.

We hypothesise that, at the time when Lake Tanganyika was divided into several paleolakes [54, 55], an ancestor of the southern lineage, which includes *T*. sp. ‘maculatus’, *T*. sp. ‘red’, *T. moorii* s.l. and *T*. sp. ‘Mpimbwe’ (Additional file 3) [38] occupied the rocky shores of a paleolake that corresponds to the modern southern subbasin. Possibly, it was the only representative of the genus present. As stenotypic littoral cichlids were shown to colonise lakeshores following a stepping stone model [56], parts of the genetic and morphological clinal variation could already have been shaped along the shores of this paleolake. At the same time, *T. brichardi* occurred in a separate paleolake further north.

Following a rise in lake level, the different paleolakes merged. This allowed *T. brichardi* to colonise the southern subbasin. As a *T. moorii*-like variety also occurs in a limited part of the central subbasin [9, 38], a similar colonisation could have taken place in the opposite direction. Currently, *Tropheus* occurs at every stretch of rocky shore of Lake Tanganyika. Such ubiquitous occurrence has likely already existed in the past and southern species of *Tropheus* were probably present at all suitable habitats during the colonisation of *T. brichardi*. Therefore, *T. brichardi* might initially have shared its habitat with a resident species. A similar situation is currently seen south of the Kipili Islands where the distribution ranges of *T. moorii* and *T. brichardi* overlap. Here, *T. moorii* occurs at the upper parts of the shoreline whereas *T. brichardi* occupies the deeper parts that are more covered in sediment, and as such comprise lower quality feeding grounds [9]. Given that, around the Kipili Islands, only *T. brichardi* is found, we hypothesise that it was able to replace a *T. moorii*-like species there.

North of the Kipili islands, hybridization between the invading *T. brichardi* and the resident *Tropheus* might have given rise to *T*. sp. ‘Mpimbwe’. Both genetic and morphological data revealed similarities between *T. brichardi* ‘Kipili’ and *T.* sp. ‘Mpimbwe’. In the PCA on AFLP fingerprints (Figs. 2,3), the latter fell intermediately between *T. brichardi* ‘Kipili’ and the other groups. Additionally, some solutions of the Structure analysis (Additional file 4) assigned *T.* sp. ‘Mpimbwe’ to the same group as *T. brichardi* ‘Kipili’, but also suggested some affinity with *T.* sp. ‘maculatus’ that belongs to the southern lineage. This signal was also retrieved in the heat map (Fig. 4) and it might reflect ancient gene flow or a shared origin dating from the time when the northeastern and northwestern shores of the subbasin were connected by lake level low stands. The morphological similarity between *T. brichardi* ‘Kipili’ and *T.* sp. ‘Mpimbwe’ is reflected by their similar values for the most important axes that describe variation in meristics (SR-PC1) and shape (PC2) (Fig. 5). Finally, the two groups also bear similar mitochondrial haplotypes [36], even though AFLP-based phylogenies assign them to different evolutionary lineages (Additional file 3) [38]. These patterns all point towards a hybrid origin of *T*. sp. ‘Mpimbwe’, or at least to a significant influx of genetic material of *T. brichardi* into the species. Konings [9] already suggested a hybrid origin for *T.* sp. ‘Mpimbwe’, although he proposed *T. annectens* Boulenger, 1900, and not *T. brichardi*, as a potential parent. For this, he referred to a phylogeny reconstruction [46] and to the juvenile colouration, which is highly similar in *T.* sp. ‘Mpimbwe’ and *T. annectens.* As the latter species is not included in our dataset, we cannot exclude this scenario. However, as *T. annectens* and *T. brichardi* occur in sympatry at the central subbasin, this scenario would also require a colonisation event from the central to the southern subbasin.

### Lake level fluctuations shaped clinal variation in southern populations of Tropheus

*Tropheus moorii* s.l., *T.* sp. ‘maculatus’, and *T.* sp. ‘red’ all belong to a single lineage that is near-endemic to the southern subbasin (Additional file 3) [38]. Hence, they most probably originated within this subbasin. The distribution of all of these species contains at least one part of the shoreline that was classified as steep (Fig. 1). Populations from such shores were less affected by changes in lake level, which mainly caused a vertical shift in distribution, and hence kept population connectivity stable [31]. Consequently, these populations could have remained separated for sufficient amounts of time. This, in combination with the stenotypic behaviour of *Tropheus* [29] explains how a parent species could have diverged allopatrically into distinct species along steep shores. We hypothesise that the currently observed sympatry between *T.* sp. ‘red’ and *T.* sp. ‘maculatus’ stems from secondary contact, after species boundaries were established.

All datasets revealed clinal variation between neighbouring populations from the shallower southernmost part of the southern subbasin. This part of the lake was dry during periods of low lake stand. Hence, the current southernmost populations could stem from source populations originating from steeper southwestern: *T.* sp. ‘red’, and southeastern shores: *T. moorii* ‘Southeast’. Alternatively, the ancestors of these populations were, at periods of low lake levels, restricted to shores that were much closer to the distributions of *T.* sp. ‘red’ and *T. moorii* ‘Southeast’, possibly allowing for gene flow. Whatever the case, repeated fluctuations in water levels would certainly have caused several extinction, colonisation and hybridisation events between these populations [29]. Although these events were most probably highly complex, their combined effects would average out in a clinal pattern as observed today, with populations from western shores being more similar to *T*. sp. ‘red’ and those from eastern shores being more similar to *T. moorii* ‘Southeast’.

An influx of genetic material stemming from *T*. sp. ‘red’ and *T. moorii* ‘South’ was also observed in *T.* sp. ‘yellow’. Both genetic data and experimental crosses showed that this variety is a natural hybrid between *T.* sp. ‘red’ and *T. moorii* ‘South’ [13]. The clinal variation observed in morphology (Fig. 5) and genetics (Figs. 2, 3, Additional file 4) agreed with this hybrid origin. All but one of the *T.* sp. ‘red’ clustered with *T.* sp. ‘yellow’ and *T. moorii* ‘South’ in the NJ tree (Additional file 3). It should be noted, however, that all but one of the *T.* sp. ‘red’ specimens used for the molecular analysis, stem from the shallow southern reaches of the species’ distribution. Hence, our model suggests that these populations too could have received some genetic material from *T. moorii* s.l., explaining why *T.* sp. ‘red’ was not supported as a distinct lineage in the NJ tree. We therefore hypothesise that the ‘true’ *T.* sp. ‘red’ populations, which stayed isolated from *T. moorii* s.l., are found along the steeper shores further north. However, only one specimen of such populations was available for the genetic study and only six were available for the morphological approach.

### Linking morphological variation with the stability of the shoreline

Lake level fluctuations have been invoked as an important factor to explain the explosive speciation of Great Lake cichlids [31, 56]. Hence, the morphological variation of these cichlids should also be interpreted in view of these events. By using the bathymetry of the shore as a proxy for the age of its resident population of *Tropheus,* we were able to compare morphological variation within old (evolutionary stable) and young (dynamic) populations. As morphological variation reflects, at least partially, genetic variation, we expected similar patterns in analyses of meristics and shape. However, whereas meristic variation was higher in populations from steep than from shallow shores, the opposite held for variation in shape. This could be explained by taking the timescale at which lake level changes occurred into account. Although one might expect older populations to be genetically more diverse, the opposite was shown to hold as, at least for the mitochondrial control region, younger populations from shallow shores contained a higher diversity [31]. This was interpreted as a signature of historic population dynamics. Indeed, whereas haplotypes can disappear in all populations through genetic drift, they are lost for good in populations from steeper shores. At shallow shores, only relatively minor fluctuations in lake level suffice to reshuffle populations, providing opportunities for admixis and exchange of haplotypes. Because variation in body shape should have some genetic basis, the same process of genetic admixis may also explain the higher intra-population variation in shape along the shallow shores. Additionally, these populations might not have occupied shallow shores long enough to have evolved a shape that is best adapted to the local conditions.

We postulate that populations from steeper shores have occupied parts of the shoreline that are close to their current habitats for much longer periods of time. As habitat influences shape in *Tropheus* [10], this could have provided them with the opportunity for local adaptation and to evolutionarily fix this body shape within the population. This explains the lower degree of variation in shape within sites, but also the higher degree of variation in shape between sites along steeper shores. Conspecific populations from steep shores can sometimes differ drastically in shape, which was, for example, the case between northern and southern populations of *T*. sp. ‘maculatus’ and *T.* sp. ‘Mpimbwe’ (Fig. 5d). Such cases also explain the high amount of overlap between inter- and intra- specific P_ST_-values. However, large differentiation in shape was not observed between populations from shallow shores, not even when more distant comparisons, e.g. between *T. moorii* ‘yellow’ and its parent groups, were made.

Populations of *Tropheus* from steep shores are more variable in meristics than those from shallow shores. Although the same evolutionary pressures worked on shape and on meristics, these could have worked on different time scales. Whereas only a minor drop in water level already drastically distorted the shallow shoreline, changes of a few hundred meters were required to have similar effects on steeper shores. However, such drops did occur when East Africa witnessed periods of local aridity [54, 55], as was demonstrated by studying drill cores from Lake Malawi [57]. Therefore, distortions of the shoreline affecting populations from the steeper shores did happen, but at a much lower frequency. Hence, we expect that similar processes that maintained elevated levels of diversity in unstable or rapidly evolving markers along the shallow shores would account for high levels of diversity in more stable characters in populations from steeper shores. As meristics are more variable than shape in populations from steeper shores, this variation could have been formed by fluctuations that occurred at a lower frequency than those that shaped variation in shape. Hence, we can interpret meristics as more evolutionary stable than characters describing shape, and therefore more taxonomically informative. Moreover, when tooth counts were omitted from the analyses, the difference in variation between steep and shallow shores was even more pronounced (not shown). This implies that tooth counts are less stable than other meristic traits. Populations from shallow shores most likely descend from lineages that, at periods of extreme low water stand, inhabited only a short stretch of coastline. Due to founder effects, a high amount of meristic variation might never have been present here. So, whereas the high variation in body shape along shallow shores could reflect admixis between closely related lineages, the high variation in meristics at steeper shores might reflect admixis between distantly related lineages, or even between different species.

The higher stability of steeper shores can also explain several instances of sympatry in *Tropheus*. Throughout the lake, such instances are never found along shallow shores. Hence, we speculate that whereas the frequent habitat disturbances along shallow shores might have caused extinctions and hybridisations, the long-term stability of steeper shores could have allowed for species coexistence. The frequent disturbances at the shallow shores, did, however, not hamper the development and maintenance of clearly distinct colour varieties. Even though the southernmost end of the lake harbours a young *Tropheus* assembly, its populations differ profoundly in colouration [9, 29]. Conversely, neighbouring populations from steeper shores sometimes differ very little in colouration. For example, *T.* sp. ‘maculatus’ contains only two distinct colour varieties: a ‘striped’ northern and a ‘blotched’ southern form, whereas dozens of colour varieties are present in *T. moorii* s.l. [9]. Lake level fluctuations caused frequent displacements of barriers to dispersal at shallow shores, which might have led to the emergence of novel colour patterns due to hybridization and drift [13]. Hence, we can speculate that chromatic differentiation emerged even faster than differentiation in shape. The evolution of distinct colour patterns could even have been accelerated by colour-mediated mate choice [36, 58]. Moreover, a certain degree of clinal variation in mate preference can also be observed as, following an anthropogenic introduction, specimens from distant populations were less likely to hybridise than specimens from nearby locations [36, 59].

Many colour varieties of *Tropheus* from steeper shores possess a pattern of clearly visible vertical bars on the flanks. In specimens from the shallow southernmost shores, this pattern is rare. Although bars are often still visible on juveniles, adults of many varieties of *T.* sp. ‘red’ and *T. moorii* s.l. lack such bars. In lacustrine cichlids, a pattern of vertical bars is present in the majority of species that occur at littoral zones with strong habitat structure, such as rocky shores [60]. This could render them less conspicuous to predators. Banded patterns are also seen in populations that are currently assigned to various species of *Tropheus*, including *T. moorii*, *T. brichardi* and *T. annectens*. This could indicate that such a pattern is plesiomorphic for the southern lineage of *Tropheus*. This plesiomorphy could also explain the high similarity between *T.* sp. ‘maculatus’ from Murago at the western and a variety of *T. moorii* collected near Wapembwe at the eastern shore. The latter variety was even labelled the “Tanzania Murago” in the aquarium trade [9]. Our AFLP scans (Figs. 2,3), however, supported the latter’s placement in *T. moorii*. Hence, unstable shorelines, which provided opportunities for hybridisation followed by colour-based mate choice, could have been favourable for the evolution of non-barred colour patterns in *Tropheus*. At these shores, the effects of sexual selection might have surpassed those of natural selection, leading to the development of non-banded patterns. Two species of *Tropheus* from other subbasins lack populations with a banded adult colouration pattern: *T. duboisi* and *T.* sp. ‘black’. The former occurs at deeper parts of the shore, where they are less vulnerable to avian predation. Most populations of the latter are found at shallow shorelines in the northern and central subbasins. Additionally, *T.* sp. ‘black’ also contains a relatively large amount of intra-specific chromatic differentiation among populations in the northern subbasin. The processes that shaped this diversity could have been highly similar to those that took place in the south. The populations of *T.* sp. ‘black’ from steep shores in the central subbasin, are again found at deeper parts of the shore, as the upper parts are occupied by a banded variety of *T. annectens*.

### The taxonomic value of different morphological characters in rock-dwelling cichlids

Both the comparison of P_ST_-values and the LOESS curves on the PC’s showed that meristics provided better support for the AFLP-based classification than variables describing shape. Nevertheless, even though the overlap between species was high for PC axes describing variation in shape, most of the bending points in the LOESS curves (Fig. 5d-f) reflected inter-specific differentiation. This indicates that although differences in shape can have a taxonomic value, they should be interpreted carefully.

Although their genetic basis remains unknown, the differences in taxonomic content between meristics and variables describing shape could reflect different rates of morphological evolution. Many of the measurements studied here relate to trophic morphology, swimming ability and stability in the water column. Hence, considerable amounts of natural selection are expected to work on them. Many meristics, however, could be considered relatively ‘neutral’. This could explain why differentiation in shape might have evolved faster than differentiation in meristics. It should be noted, however, that shape is also prone to a significant deal of plasticity in *Tropheus* [61]. In our analyses, tooth counts also behaved more as variables describing shape. Hence, they can be considered less taxonomically reliable, and less evolutionarily stable than other meristic traits in *Tropheus*.

As our analyses only included southern representatives of *Tropheus,* the observed patterns might not hold for other cichlids from Lake Tanganyika, or not even for all populations of *Tropheus*. However, a close link between meristic data and taxonomy, and a weaker link between shape and taxonomy, was seen in several studies. For example, in an intra-specific comparison of populations of *T. duboisi*, analyses of body shape completely separated groups for which meristic values largely overlapped [12]. Conversely, in a revision of the closely-related *Pseudosimochromis* Nelissen 1978 [19], meristics could be used to separate all species whereas measurements failed to do so. However, both measurements and meristics revealed similar amounts of intra-specific differentiation in the geographically strongly structured *P. babaulti* (Pellegrin, 1927). The different taxonomic content of meristics and measurements also holds for more distantly-related Lake Tanganyika cichlid genera. In an inter-specific study on *Ophthalmotilapia* Pellegrin, 1904 [62], meristic data almost completely separated the four species, whereas overlap was much larger for measurements. In *Neolamprologus niger* (Poll, 1956) [63], on the other hand, both measurements and meristics revealed similar amounts of intra-specific differentiation.

The question remains to what extent our results can be extrapolated to the study of littoral cichlids of Lake Malawi and of the Lake Victoria superflock. Although the *Tropheus* radiation belongs to the modern haplochromines *sensu* *lato* [64], the lineage that also radiated in the other Great Lakes, it is significantly older than similar radiations from these other lakes [7, 65]. Moreover, notwithstanding the profound effects of lake level fluctuations on cichlids from Lake Tanganyika, these were smaller than the effects of similar fluctuations in lakes Victoria and Malawi. Whereas, in Lake Tanganyika, large sections of shoreline are situated close to deep (> 500 m) parts of the lake, this is only the case for a small fraction of the Malawi shoreline [66]. Hence, not only is the Malawi radiation of a much younger age [67], but also do most of its littoral cichlids occupy shallow, unstable shores. The situation is even more extreme in Lake Victoria, which has a maximal depth of 80 m and which might have dried out completely as recently as 14.7 KYA [68].

Given its young age and high speciation rates [69], meristic traits are often of little use to distinguish closely-related species from the Lake Victoria superflock [70, 71]. In the more mature Lake Malawi radiation, meristics sometimes contain information to delineate species, whereas, in other cases, this only holds for measurements, e.g. [72]. Although this supports our treatment of meristics as slowly evolving, evolutionarily stable traits, it also implies a need to rely on differences in body shape, which we showed to be problematic. An example hereof is provided by the sympatric species *Nimbochromis livingstonii* (Günther, 1893) and *N. polystigma* (Regan, 1922), which did not differ in meristic traits. When looking only at samples from a restricted part of the lake, both species could be separated using measurements. Yet, when specimens from the entire lake were compared, intra-specific variation in measurements surpassed inter-specific differences [73].

The same climatic events that led to the inundation of the shallow southern parts of Lake Tanganyika also transformed Lake Malawi from a small into a large lake [29, 66, 67]. These events triggered the evolution of allopatric colour varieties in *Tropheus* from the shallow parts of Lake Tanganyika, and in rock dwelling ‘Mbuna’ species from Lake Malawi. Therefore, the morphological differentiation between ecologically similar Mbuna populations can be expected to be comparable to that between southern *Tropheus* populations. Indeed, a study on one of these Mbuna species, the algae scraper *Maylandia zebra* (Boulenger, 1899), also revealed considerable differentiation in measurements and meristics [74]. As values between most populations overlapped, the resulting pattern was reminiscent of that observed in southern populations of *Tropheus*. Morphological differences were further also observed between native and translocated populations of *Maylandia aurora* (Burgess, 1976) and *M. callainos* (Stauffer & Hert, 1992) [72]. As these populations were only separated for 20 years, such differences cannot be interpreted as being inter-specific, indicating the need to interpret them carefully.

The high degree of chromatic differentiation in *Tropheus* from the shallow southernmost part of Lake Tanganyika is reminiscent to the situation in many Mbuna species. In the latter, closely-related allopatric species often differ profoundly in (male) colouration. As colour patterns are important in mate recognition [75], chromatic differences have been argued to be more informative than morphological criteria for species delineation in Mbuna [76]. Hence, allopatric populations with highly similar morphology but different colour patterns are often treated as different species. If a similar criterion would be applied to *Tropheus*, this could justify the elevation to the species rank of many colour varieties, especially in *T. moorii* and *T.* sp. ‘red’ from the shallow shores. Although a certain amount of assortative mating was observed between these varieties, this did not necessitate treating them as different species [77]. Here, we argue that such a criterion for species delineation cannot be applied to *Tropheus*. If it would be applied for the southernmost colour varieties, it would render the classification of populations from steeper parts of the shoreline problematic. At steep shores, inter-population genetic and morphological differentiation is often more pronounced, in spite of smaller chromatic differentiation, than at shallow shores. This problem is less present in classifying Lake Malawi Mbuna as, due to the morphology of the lake, almost all populations can be considered relatively recent. Hence, there, we don’t necessarily expect a similar conflict between classifications based on chromatic, morphological or genetic characters.

## Conclusions

Above, we argued that the influences of historical events should be taken into account when classifying taxa with inherently complex evolutionary histories, such as the representatives of *Tropheus*. However, when an evolutionary species concept is adhered to, also the future stability of a species should be taken into account. When evaluating this concept, Wiley and Mayden [78, 79] considered a species as: “an entity composed of organisms which maintains its identity from other such entities through time and over space, and which has its own independent evolutionary fate and historical tendencies.” This definition also implies that species should have the potential for a particular historical trajectory that could allow them to maintain their identity from other species. Hence, expected future events might also be taken into consideration when delineating species. Future lake level changes will continue to cause admixture between populations of *Tropheus* from the southernmost shallow shores of Lake Tanganyika, whereas populations from steep shores will remain relatively isolated from each other for much longer periods of time. The same reasoning cannot be applied to the radiations of lakes Victoria and Malawi. Hence, besides having a shorter evolutionary history, littoral cichlid species from these lakes should also be seen as having a shorter evolutionary future. It has been argued before that the large discrepancy in species richness between the radiations of the different Great Lakes can be partially explained by the use of different cut-off values between inter- and intra-specific differentiation. Here we argue that intrinsic properties of the lakes themselves, and of the cichlid flocks they harbour, necessitate the use of standards that work best for ‘most of the radiation’. Hence, given that most of Lake Tanganyika consists of steep and stable shorelines, stenotypic cichlids occupying the shallow southernmost part of the lake might be intrinsically difficult to classify, when ill-adapted standards are being used.

## Additional files

Additional file 1: Tables (.docx with: 1) list of material and sampling sites, 2) morphological parameters studied, 3) results of the PCA on the merictic and 4) on the measurement data. (DOCX 50 kb)

Additional file 2: MDS plot on 108 AFLP fingerprints performed with Jaccard similarity indices. The northernmost specimen of *T.* sp. ‘red’ (Kikoti, loc. 4) is visualised separately as it is not included in the convex hull with the other specimens of *T.* sp. ‘red’. (JPG 99 kb)

Additional file 3: AFLP-based Neighbour Joining tree of 108 southern specimens of *Tropheus*. Three *T. duboisi* specimens were used as outgroup, statistical support was estimated by performing 1000 bootstrap replicates. (PDF 8 kb)

Additional file 4: Output of cluster analysis performed in Structure. Groups are indicated as they occur along the shoreline with 1: *T.* sp. ‘maculatus’, 2: *T.* sp. ‘red’, 3: *T. moorii* ‘yellow’, 4: *T. moorii* ‘South’, 5: *T. moorii* ‘Southeast’, 6: *T. brichardi* ‘Kipili’, 7: *T.* sp. ‘Mpimbwe’. The northernmost specimen of *T.* sp. ‘red’ (Kikoti, loc. 4) is represented by the first sample of group 2, even though it appears within the distribution range of group 1. (PDF 317 kb)

Additional file 5: Estimates of delta K and L (K) given by Structure. (PDF 2935 kb)

Additional file 6: Morphological variation vs. geographical distance along the shoreline for the individual meristics and measurements. Measurements were expressed as percentages of head (for measurements taken on the head) or standard length, raw data were shown for meristics and for SL; the distance was taken along the shoreline (in km) starting from the northwesternmost locality. LOESS curves were calculated with a fixed smoothening parameter of 0.2. As this resulted in a straight line for ASp, 0.1 was used instead (*). (PDF 4487 kb)
