# Supplementary material for: Delineating species along shifting shorelines: Tropheus (Teleostei, Cichlidae) from the southern subbasin of Lake Tanganyika
Source: Front Zool. 2018 Nov 13;15:42. doi: 10.1186/s12983-018-0287-4 (PMC6234679; doi:10.1186/s12983-018-0287-4)
Supplement: Supplementary file 1 — Tables (.docx with: 1) list of material and sampling sites, 2) morphological parameters studied, 3) results of the PCA on the merictic and 4) on the measurement data. (DOCX 50 kb) [file 12983_2018_287_MOESM1_ESM.docx]

**Additional file 1: Tables**

**Additional Table 1.** Summary of examined specimens, assigned to the groups as used in the analyses. Localities are numbered counter clockwise along the southwestern, southern and southeastern lakeshore. Localities are divided into steep (1) or shallow (0) shore types (ST), G and M indicate the number of specimens used for the genetic and morphological studies, S and E: latitude and longitude of the catch localities, km: geographic distance between locations in kilometres, taken along the lakeshore and starting from the northwesternmost locality. For specimens collected during the T92 and T95 expeditions, locality numbers as used in the expedition logs are given. For four locations, all (1, 4, 93) or some (3) of the specimens used for AFLP were also included in the morphological study.

| loc. | group | Locality | G | M | ST | S | E | km |
| --- | --- | --- | --- | --- | --- | --- | --- | --- |
| 1 | *T*. sp. ‘maculatus’ | Migenzi | 2 | 8 | 1 | 7.08 | 29.89 | 0.00 |
| 2 | *T*. sp. ‘maculatus’ | Mufazi |  | 19 | 1 | 7.09 | 29.91 | 2.75 |
| 3 | *T*. sp. ‘maculatus’ | Kyanza | 2 | 10 | 1 | 7.11 | 29.98 | 10.29 |
| 4 | *T*. sp. ‘maculatus’ | Kikoti | 3 | 8 | 1 | 7.19 | 30.07 | 23.66 |
| 4 | *T*. sp. ‘red’ | Kikoti | 1 | 1 | 1 | 7.19 | 30.07 | 23.66 |
| / | *T*. sp. ‘maculatus’ | Obtained via aquarium trade | 3 |  | 1 | / | / |  |
| 5 | *T*. sp. ‘maculatus’ | Tembwe |  | 7 | 1 | 7.24 | 30.12 | 31.61 |
| 6 | *T*. sp. ‘maculatus’ | Zongwe? | 1 |  | 1 | 7.30 | 30.13 | 39.22 |
| 7 | *T*. sp. ‘maculatus’ | 2me crique N de Masanza |  | 10 | 1 | 7.39 | 30.19 | 49.93 |
| 8 | *T*. sp. ‘maculatus’ | Mwerasi, stat 319 |  | 3 | 1 | 7.43 | 30.19 | 54.86 |
| 9 | *T*. sp. ‘maculatus’ | Kapampa | 1 |  | 1 | 7.50 | 30.20 | 62.73 |
| 10 | *T*. sp. ‘maculatus’ | Kibushi, Congo |  | 1 | 1 | 7.70 | 30.23 | 84.74 |
| 11 | *T*. sp. ‘maculatus’ | Tumpa |  | 1 | 1 | 7.71 | 30.23 | 85.85 |
| 12 | *T*. sp. ‘maculatus’ | Kasenga |  | 1 | 1 | 7.73 | 30.25 | 89.57 |
| 12 | *T*. sp. ‘red’ | Kasenga |  | 1 | 1 | 7.73 | 30.25 | 89.57 |
| 13 | *T*. sp. ‘red’ | Kalo |  | 1 | 1 | 7.80 | 30.28 | 97.02 |
| 14 | *T*. sp. ‘red’ | Kamakonde, Congo |  | 1 | 1 | 7.88 | 30.31 | 107.36 |
| 15 | *T*. sp. ‘red’ | Lunangwa |  | 1 | 1 | 7.89 | 30.32 | 108.46 |
| 16 | *T*. sp. ‘red’ | Mvua, mouillage, rive nord |  | 1 | 1 | 8.11 | 30.55 | 143.41 |
| 17 | *T*. sp. ‘red’ | At the border between Zambia and Zaire |  | 2 | 0 | 8.22 | 30.57 | 156.59 |
| 18 | *T*. sp. ‘red’ | Moliro, stat 217 | 4 | 1 | 0 | 8.24 | 30.58 | 158.70 |
| / | *T*. sp. ‘red’ | Chisanze, obtained via aquarium trade | 4 |  | 0 | / | / |  |
| 19 | *T*. sp. ‘red’ | Cap Chipimbi |  | 8 | 0 | 8.30 | 30.57 | 166.27 |
| 20 | *T*. sp. ‘red’ | Chimba | 4 |  | 0 | 8.32 | 30.53 | 170.64 |
| 21 | *T*. sp. ‘red’ | Katete | 4 | 7 | 0 | 8.34 | 30.51 | 173.48 |
| 22 | *T*. sp. ‘red’ | Chisiki, T95 -10 |  | 10 | 0 | 8.34 | 30.50 | 174.86 |
| 23 | *T*. sp. ‘red’ | Mossi |  | 8 | 0 | 8.41 | 30.46 | 183.79 |
| 24 | *T*. sp. ‘red’ | Ndole | 4 |  | 0 | 8.48 | 30.45 | 190.29 |
| 25 | *T*. sp. ‘red’ | Cape Kachese, T95 -11 | 3 | 11 | 0 | 8.49 | 30.48 | 192.83 |
| 26 | *T*. sp. ‘red’ | Nsumbu village, T95 -9 |  | 2 | 0 | 8.52 | 30.48 | 196.14 |
| 27 | *T*. *moorii ‘*yellow’ | Nkamba Bay, T95 -12 |  | 12 | 0 | 8.52 | 30.58 | 206.82 |
| 28 | *T*. *moorii ‘*yellow’ | Sumbu Kamba Bay |  | 8 | 0 | 8.53 | 30.57 | 209.24 |
| 29 | *T*. *moorii ‘*yellow’ | Chilanga | 4 | 8 | 0 | 8.56 | 30.62 | 215.57 |
| / | *T*. *moorii ‘*yellow’ | Ilangi, obtained via aquarium trade | 4 |  | 0 | 8.53 | 30.63 | 216.99 |
| 30 | *T*. *moorii ‘*yellow’ | Linagu | 4 | 8 | 0 | 8.53 | 30.64 | 218.93 |
| 31 | *T*. *moorii ‘*yellow’ | Kasaba bay, T95 -13 |  | 8 | 0 | 8.52 | 30.65 | 221.14 |
| 32 | *T*. *moorii ‘*yellow’ | Kasaba Bay |  | 19 | 0 | 8.52 | 30.70 | 226.89 |
| 33 | *T*. *moorii ‘*yellow’ | Cap Kabayeye, à l'est de Kasaba bay |  | 8 | 0 | 8.53 | 30.73 | 231.01 |
| 34 | *T*. *moorii* ‘South’ | Chaitika Point, T95 -6 | 4 | 4 | 0 | 8.57 | 30.79 | 239.01 |
| 35 | *T*. *moorii* ‘South’ | Misepa, Zambia |  | 1 | 0 | 8.59 | 30.80 | 241.09 |
| 36 | *T*. *moorii* ‘South’ | Cap Chaitika |  | 5 | 0 | 8.60 | 30.82 | 243.53 |
| 37 | *T*. *moorii* ‘South’ | Nakaku village | 4 | 13 | 0 | 8.68 | 30.90 | 255.71 |
| 38 | *T*. *moorii* ‘South’ | Mupapa |  | 5 | 0 | 8.68 | 30.90 | 255.71 |
| 39 | *T*. *moorii* ‘South’ | Kaku, South |  | 4 | 0 | 8.68 | 30.91 | 256.56 |
| 40 | *T*. *moorii* ‘South’ | Tumbi |  | 8 | 0 | 8.70 | 30.92 | 259.39 |
| 41 | *T*. *moorii* ‘South’ | Funda | 4 |  | 0 | 8.77 | 30.98 | 270.43 |
| 42 | *T*. *moorii* ‘South’ | Katoto (A1) (N) | 4 | 11 | 0 | 8.79 | 31.02 | 273.94 |
| 43 | *T*. *moorii* ‘South’ | Katoto S | 4 |  | 0 | 8.80 | 31.02 | 274.50 |
| / | *T*. *moorii* ‘South’ | Chiseketi, obtained via aquarium trade | 4 |  | 0 | / | / |  |
| 44 | *T*. *moorii* ‘South’ | Kombe, Zambia |  | 3 | 0 | 8.80 | 31.02 | 274.50 |
| 45 | *T*. *moorii* ‘South’ | Tanganyika Lodge, T95 -1 | 3 | 8 | 0 | 8.78 | 31.08 | 281.68 |
| 46 | *T*. *moorii* ‘South’ | Mpulungu, Jetty, Lake Tanganyika |  | 8 | 0 | 8.77 | 31.12 | 285.99 |
| 47 | *T*. *moorii* ‘South’ | Kinyamkola |  | 4 | 0 | 8.77 | 31.12 | 285.99 |
| 48 | *T*. *moorii* ‘South’ | Kasakalawe 2 Zambia | 4 | 1 | 0 | 8.70 | 31.07 | 295.29 |
| 49 | *T*. *moorii* ‘South’ | Mbita island | 4 | 8 | 0 | 8.75 | 31.09 | 289.76 |
| 50 | *T*. *moorii* ‘South’ | Mutondwe Isl (Crocodile Isl) |  | 1 | 0 | 8.72 | 31.12 | 300.80 |
| 51 | *T*. *moorii* ‘South’ | Crocodile Island, T95 -29 |  | 1 | 0 | 8.70 | 31.12 | 302.60 |
| 52 | *T*. *moorii* ‘South’ | Kasenga Point, T95 -2 |  | 12 | 0 | 8.73 | 31.13 | 305.33 |
| 53 | *T*. *moorii* ‘South’ | Wonzye Point (95), T95 -3 |  | 3 | 0 | 8.73 | 31.13 | 305.38 |
| 54 | *T*. *moorii* ‘South’ | Wonzye Point (T11) |  | 8 | 0 | 8.72 | 31.14 | 306.17 |
| 55 | *T*. *moorii* ‘South’ | Muzumwa |  | 9 | 0 | 8.70 | 31.20 | 313.37 |
| 56 | *T*. *moorii* ‘South’ | Isanga bay, T95 -5 |  | 10 | 0 | 8.66 | 31.19 | 318.61 |
| 57 | *T*. *moorii* ‘South’ | Kalambo Lodge |  | 8 | 0 | 8.62 | 31.20 | 322.35 |
| 58 | *T*. *moorii* ‘South’ | Kapele, T95 -28 |  | 10 | 0 | 8.58 | 31.17 | 328.21 |
| 59 | *T*. *moorii* ‘South’ | Kasisi, T95-27 |  | 2 | 0 | 8.55 | 31.17 | 330.75 |
| 60 | *T*. *moorii* ‘Southeast’ | Kasanga, T9-25 | 2 | 16 | 0 | 8.43 | 31.15 | 344.54 |
| 61 | *T*. *moorii* ‘Southeast’ | Malasa Island, Tanzania |  | 10 | 1 | 8.21 | 30.95 | 377.51 |
| 62 | *T*. *moorii* ‘Southeast’ | Kala bay, T95- 22 | 2 | 12 | 1 | 8.15 | 30.97 | 385.29 |
| 63 | *T*. *moorii* ‘Southeast’ | Wampembwe, T95- 21 | 2 | 1 | 1 | 8.01 | 30.89 | 403.31 |
| / | *T*. *moorii* ‘Southeast’ | near Wampembwe, obtained via aquarium trade | 6 |  | 1 | / | / |  |
| 64 | *T*. *moorii* ‘Southeast’ | Fulwe |  | 7 | 1 | 7.95 | 30.82 | 412.70 |
| 65 | *T*. *moorii* ‘Southeast’ | Lwili Island, Tanzania |  | 5 | 1 | 7.85 | 30.78 | 424.93 |
| 66 | *T*. *moorii* ‘Southeast’ | Msamba Bay, T95-20 |  | 11 | 1 | 7.84 | 30.79 | 425.93 |
| 67 | *T*. *moorii* ‘Southeast’ | Musamba |  | 14 | 1 | 7.83 | 30.78 | 427.75 |
| 68 | *T*. *moorii* ‘Southeast’ | Ninde, T95 -19 |  | 7 | 1 | 7.67 | 30.72 | 447.15 |
| 69 | *T*. *moorii* ‘Southeast’ | Mtosi | 2 | 12 | 1 | 7.59 | 30.64 | 458.94 |
| 70 | *T*. *brichardi* ‘Kipili’ | Twiyu 1 Tanzania |  | 1 | 1 | 7.59 | 30.63 | 460.40 |
| 71 | *T*. *moorii* ‘Southeast’ | Namansi | 5 |  | 1 | 7.53 | 30.60 | 467.86 |
| 72 | *T*. *brichardi* ‘Kipili’ | Kunwe/Kipili/Tanzania |  | 1 | 1 | 7.50 | 30.58 | 471.01 |
| 73 | *T*. *brichardi* ‘Kipili’ | East of Ngosa Point, T92 -6 |  | 1 | 1 | 7.49 | 30.57 | 473.04 |
| 74 | *T*. *brichardi* ‘Kipili’ | Punda Point, T95 -15 |  | 3 | 1 | 7.45 | 30.60 | 477.71 |
| 75 | *T*. *brichardi* ‘Kipili’ | Ulwile Island, S shore, S of Nkamba Hil, T92-5 |  | 7 | 1 | 7.48 | 30.57 | 481.24 |
| 76 | *T*. *brichardi* ‘Kipili’ | Ulwile Island, N shore, T92-4 | 2 | 7 | 1 | 7.46 | 30.57 | 483.49 |
| 77 | *T*. *brichardi* ‘Kipili’ | Nvuna Island, NE shore, T92 -7 |  | 5 | 1 | 7.44 | 30.55 | 486.90 |
| 78 | *T*. *brichardi* ‘Kipili’ | Nvuna Island, NW shore, T92 -8 |  | 1 | 1 | 7.44 | 30.54 | 487.31 |
| 79 | *T*. *brichardi* ‘Kipili’ | Kerenge Island, T95 -17 |  | 2 | 1 | 7.40 | 30.56 | 491.92 |
| 80 | *T*. *brichardi* ‘Kipili’ | Manda Island, N shore, T92 -16 |  | 6 | 1 | 7.39 | 30.56 | 492.62 |
| 81 | *T*. *brichardi* ‘Kipili’ | Nkondwe Island, S shore, T92 -9 |  | 23 | 1 | 7.38 | 30.55 | 495.13 |
| 82 | *T*. *brichardi* ‘Kipili’ | Mwsa Bay N end, T92 -15 |  | 1 | 1 | 7.35 | 30.60 | 502.06 |
| 83 | *T*. *brichardi* ‘Kipili’ | Kalanswi Bay, S part, T92 -14 |  | 13 | 1 | 7.29 | 30.59 | 508.28 |
| 84 | *T*. *brichardi* ‘Kipili’ | Kampemba N shore, T92 -12 |  | 14 | 1 | 7.18 | 30.52 | 523.37 |
| 85 | *T*. sp. ‘Mpimbwe’ | Mpimbwe Hills, S shore, 1st bay E of T92-10, T92-11 |  | 12 | 1 | 7.14 | 30.51 | 528.06 |
| 86 | *T*. sp. ‘Mpimbwe’ | Mpimbwe Hills, S shore, T92 -10 |  | 3 | 1 | 7.14 | 30.50 | 528.80 |
| 87 | *T*. sp. ‘Mpimbwe’ | Cape Mpimbwe (one via aquarium trade) | 2 |  |  | 7.14 | 30.51 | 529.38 |
| 88 | *T*. sp. ‘Mpimbwe’ | Mpimbwe Hills, Shashete Bay, N part, T92 -19 |  | 6 | 1 | 7.12 | 30.50 | 530.47 |
| 89 | *T*. sp. ‘Mpimbwe’ | Cap Mpimbwe/Kol |  | 2 | 1 | 7.11 | 30.51 | 531.54 |
| 90 | *T*. sp. ‘Mpimbwe’ | Utinta Island, Tanzania |  | 3 | 0 | 7.12 | 30.52 | 533.23 |
| 91 | *T*. sp. ‘Mpimbwe’ | just south of Kasinde, T92 -20 |  | 1 | 0 | 7.10 | 30.55 | 537.55 |
| 92 | *T*. sp. ‘Mpimbwe’ | N of Mkombe, T92 -21 |  | 8 | 0 | 6.97 | 30.57 | 552.67 |
| 93 | *T*. sp. ‘Mpimbwe’ | a few km S of Karema, T92 -22 | 2 | 2 | 0 | 6.90 | 30.49 | 564.11 |
| 94 | *T*. sp. ‘Mpimbwe’ | S of Karema, T92 -23 |  | 12 | 0 | 6.84 | 30.45 | 571.91 |

**Additional Table 2: Meristics and measurments collected in this study.**

| **meristics** | **Description** |
| --- | --- |
| **DSp** | number Dorsal Spines |
| **DSR** | number Dorsal Soft Rays |
| **ASp** | number Anal Spines |
| **ASR** | number Anal Soft Rays |
| **Pect** | number of Pectoral Rays |
| **ULat** | number of scales in the Upper Lateral Line |
| **LLat** | number of scales in the Lower Lateral Line |
| **Long** | number of scales in the Longitudinal Line; when a minute lateral line scale, shorter than half of the previous scale, was present before the articulation, this was counted as 0.5 |
| **LChS** | number of Lower Scale rows on the Cheek: the first post-ocular scale that does not belong to any scale row containing more than one scale is counted as the last row |
| **TChS** | Total number of Scale rows on the Cheek: the lower scale rows plus all post-ocular scales |
| **UGR** | number of Upper Gill Rakers |
| **LGR** | number of Lower Gill Rakers |
| **ULT** | number of Upper Lateral (tri- and unicuspid) Teeth |
| **UBT** | number of Upper Bicuspid Teeth in the outer teeth row |
| **LBT** | number of Lower Bicuspid Teeth in the outer teeth row |
| **CP** | number of scales around the Caudal Peduncle |
| **measurements** |  |
| **SL** | Standard Length |
| **LaD** | Lachrymal Depth |
| **SnL** | Snout Length: distance from the corner of the bony orbit to the tip of the upper jaw |
| **LJL** | Lower Jaw Length |
| **PPL** | Premaxillary Processus Length |
| **ChD** | Cheek Depth |
| **ED** | Eye Diameter |
| **IOW** | Inter-Orbital Width |
| **HW** | head width |
| **HL** | Head Length |
| **LJF** | Lower Jaw width Front: width of the lower jaw measured at the lateral-most bicuspid teeth |
| **UJF** | Upper Jaw width Front; width of the upper jaw measured at the lateral-most bicuspid teeth |
| **UJB** | Upper Jaw width Back; width of the upper jaw measured at the lateral-most unicuspid teeth |
| **LJB** | Lower Jaw width Back: width of the lower jaw measured behind the articulation of the jaws |
| **BD** | Body Depth |
| **DFB** | Dorsal Fin Base |
| **AFB** | Anal Fin Base |
| **PrD** | Pre Dorsal distance |
| **PrP** | Pre Pectoral distance |
| **PrV** | Pre Ventral distance |
| **PrA** | Pre Anal distance |
| **CPL** | Caudal Peduncle Length, measured between the vertical lines through the caudal point of the dorsal fin insertion and through the caudal border of the hypurals |
| **CPD** | Caudal Peduncle Depth |

**Additional table 3.** Loadings and explained variance of the PCA performed on 15 meristics of 570 specimens. Linear regression is calculated between the PC’s and SL with a: slope, b: intercept and p: probability that the PC is uncorrelated with SL. The most important loadings for each PC are indicated in bold. Full trait names are listed in Table S2.

| Trait | PC1 | PC2 | PC3 |
| --- | --- | --- | --- |
| DSp | **-0.3708** | 0.1579 | **0.3969** |
| DSR | **0.3809** | 0.1963 | -0.1898 |
| ASp | **-0.3557** | -0.1376 | **0.3412** |
| ASR | **0.3180** | 0.2691 | -0.2765 |
| Pect | 0.0012 | -0.0388 | -0.0306 |
| ULat | 0.0180 | 0.3562 | 0.2946 |
| LLat | 0.1868 | 0.1053 | 0.2472 |
| Long | 0.1001 | 0.3207 | **0.4376** |
| LChS | 0.1286 | **0.4590** | 0.0260 |
| TChS | 0.0445 | **0.4179** | 0.1101 |
| LGR | 0.1601 | -0.2059 | 0.2165 |
| UGR | -0.1428 | 0.0147 | -0.0806 |
| ULT | -0.0868 | -0.0338 | -0.0202 |
| UBT | **0.4370** | -0.2960 | 0.3192 |
| LBT | **0.4260** | -0.2934 | 0.3237 |
| % variance | 16.5240 | 14.583 | 12.439 |
| a | 0.0527 | -0.0210 | 0.1255 |
| b | -3.9456 | 0.0056 | -9.4004 |
| p (a=0) | 3.34 10^-19^ | 0.0002 | 5.93 10^-25^ |

**Additional table S4.** Loadings of the variables on the first four PC of the PCA on the covariance matrix of 23 log-transformed measurements taken on 570 specimens and their explained variance. The most important loadings for each PC (except for PC1) are indicated in bold. Full trait names are listed in Table S2.

| Trait | PC1 | PC2 | PC3 | PC4 |
| --- | --- | --- | --- | --- |
| LaD | 0.2589 | 0.0934 | -0.0666 | -0.1205 |
| SnL | 0.2358 | -0.0105 | -0.0070 | 0.1875 |
| LJL | 0.1891 | 0.1193 | -0.2325 | -0.1218 |
| PPL | 0.1796 | 0.1860 | -0.1696 | -0.1245 |
| ChD | 0.2599 | 0.0996 | -0.1621 | 0.0190 |
| ED | 0.1249 | 0.1217 | -0.1541 | 0.0469 |
| IOW | 0.2293 | 0.1273 | -0.0016 | -0.1501 |
| HW | 0.2016 | -0.0373 | -0.0749 | -0.0818 |
| HL | 0.1938 | 0.0875 | -0.0769 | 0.0733 |
| LJF | 0.2354 | **-0.3692** | 0.1089 | -0.0131 |
| UJF | 0.2365 | **-0.3962** | 0.1051 | -0.0093 |
| UJB | 0.2520 | **-0.3981** | 0.0225 | -0.0483 |
| LJB | 0.2604 | **-0.4873** | -0.0891 | -0.1302 |
| SL | 0.1889 | 0.1199 | 0.0699 | 0.0772 |
| BD | 0.1921 | 0.1000 | -0.0766 | -0.1718 |
| DFB | 0.1917 | 0.1829 | 0.0118 | -0.2189 |
| AFB | 0.1935 | 0.2206 | 0.0550 | -0.2462 |
| PrD | 0.1887 | 0.1252 | -0.1117 | -0.0645 |
| PrP | 0.1831 | 0.0553 | -0.0145 | **0.5049** |
| PrV | 0.1853 | 0.0557 | -0.0118 | **0.6120** |
| PrA | 0.1866 | 0.1287 | 0.0154 | 0.2732 |
| CPL | 0.1898 | 0.1744 | **0.8899** | -0.0635 |
| CPD | 0.1780 | 0.1666 | -0.0292 | -0.0839 |
| % variance | 92.492 | 2.3905 | 0.9183 | 0.8850 |
