# Supplementary figures and images for: Delineating species along shifting shorelines: Tropheus (Teleostei, Cichlidae) from the southern subbasin of Lake Tanganyika
Source: Front Zool. 2018 Nov 13;15:42. doi: 10.1186/s12983-018-0287-4 (PMC6234679; doi:10.1186/s12983-018-0287-4)

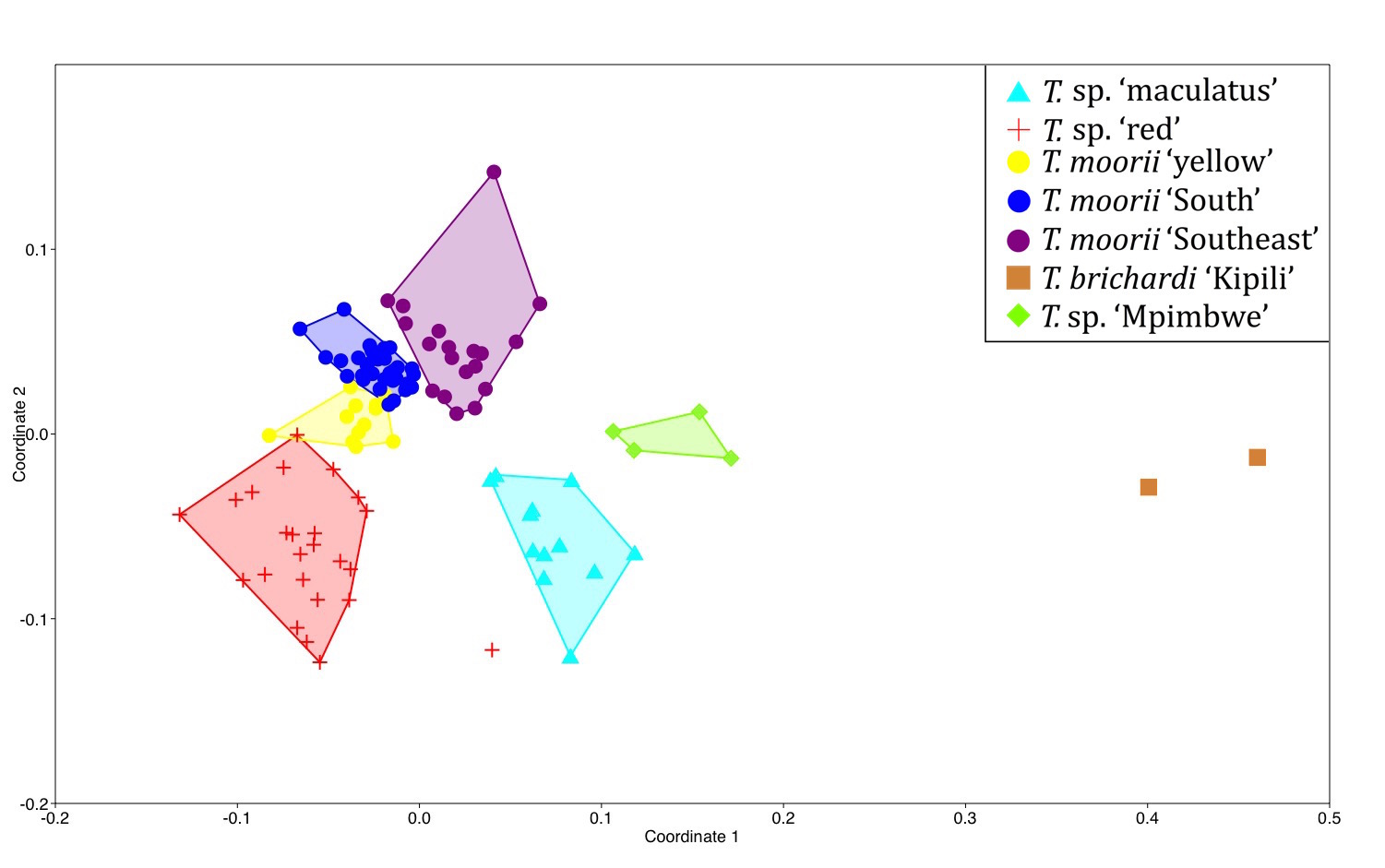

Supplement: Supplementary file 2 — MDS plot on 108 AFLP fingerprints performed with Jaccard similarity indices. The northernmost specimen of T. sp. ‘red’ (Kikoti, loc. 4) is visualised separately as it is not included in the convex hull with the other specimens of T. sp. ‘red’. (JPG 99 kb) [file 12983_2018_287_MOESM2_ESM.jpg]

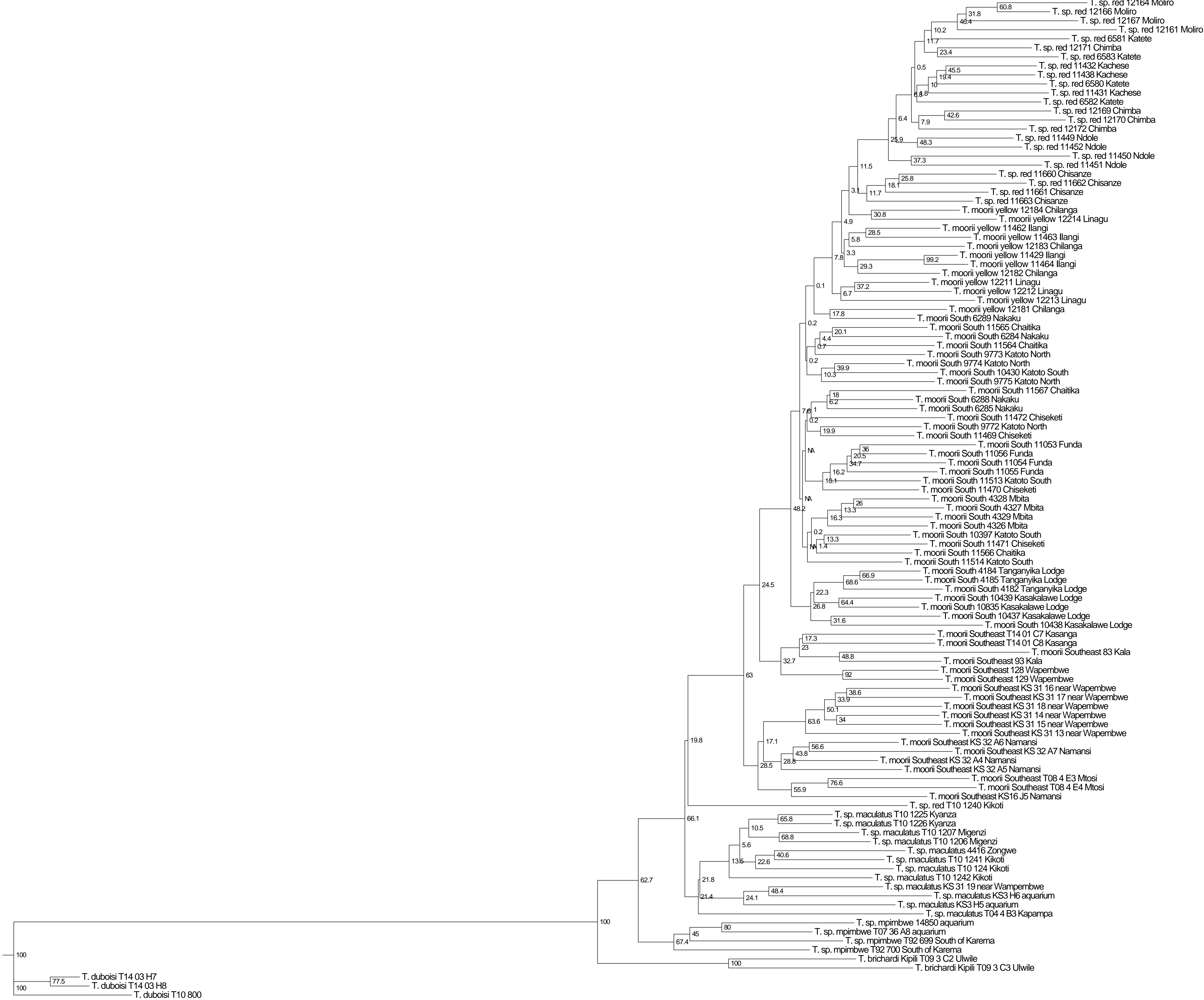

Supplement: Supplementary file 3 — AFLP-based Neighbour Joining tree of 108 southern specimens of Tropheus. Three T. duboisi specimens were used as outgroup, statistical support was estimated by performing 1000 bootstrap replicates. (PDF 8 kb) [file 12983_2018_287_MOESM3_ESM.pdf]

$$\text{Delta K} = \text{mean}(|L''(K)|) / \text{sd}(L(K))$$

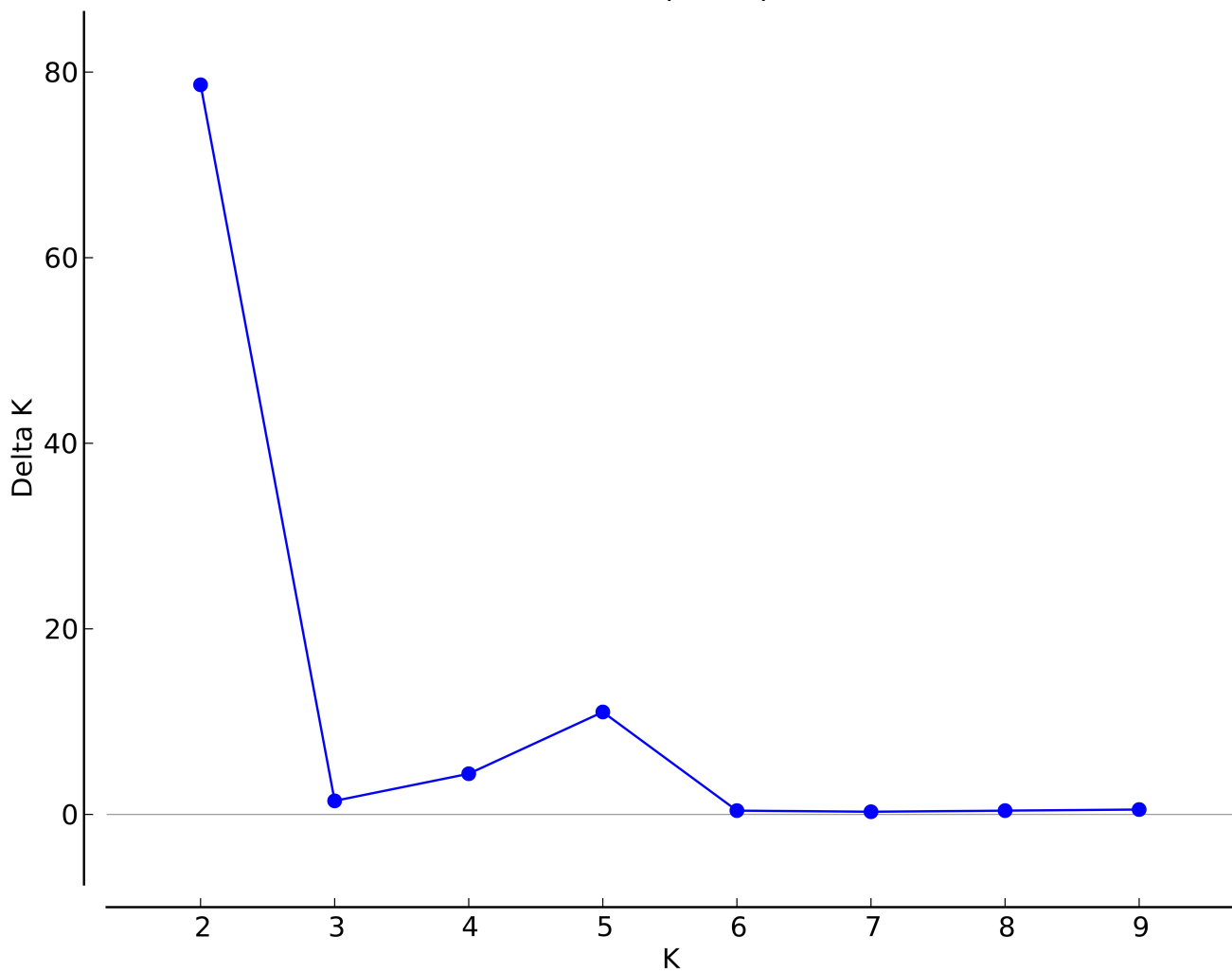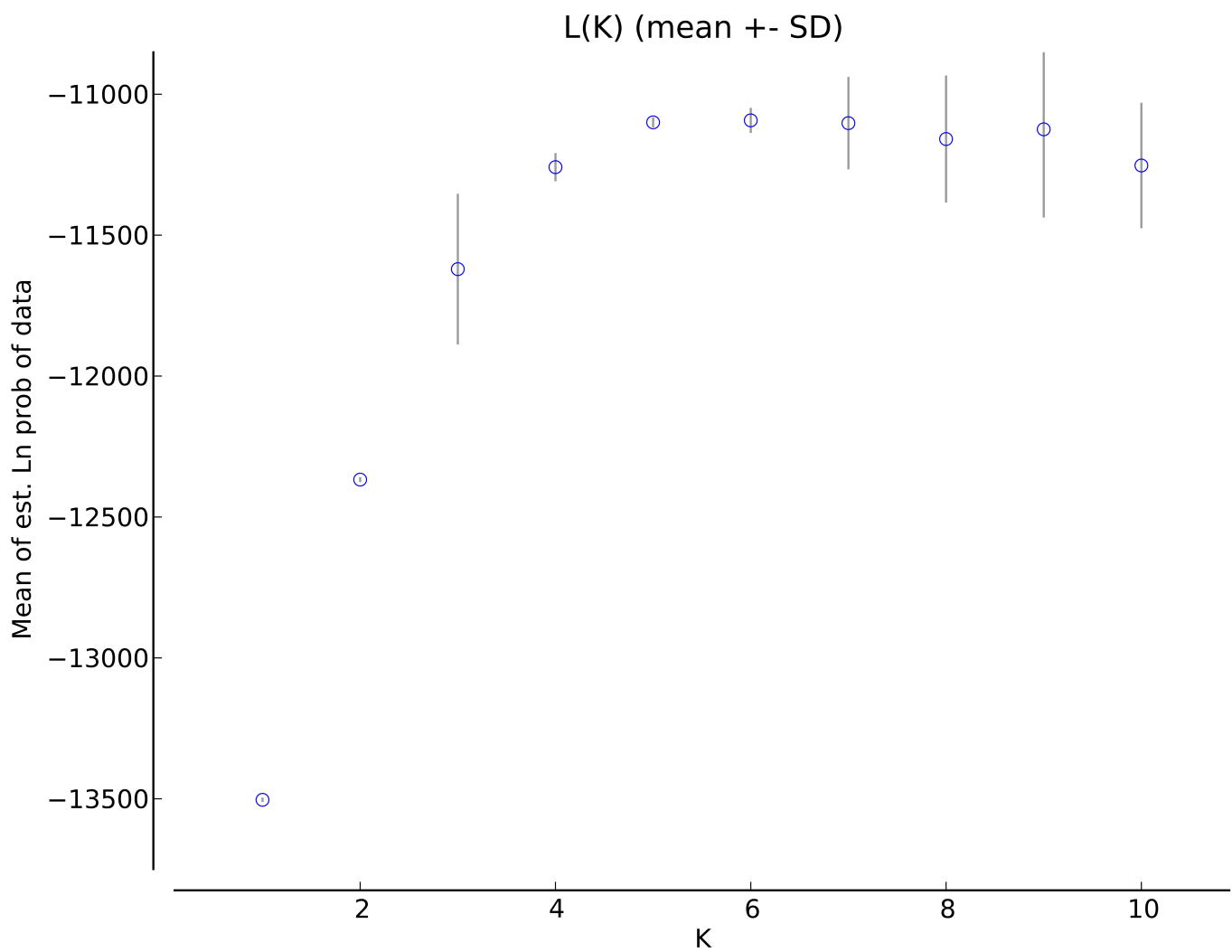

Supplement: Supplementary file 5 — Estimates of delta K and L (K) given by Structure. (PDF 2935 kb) [file 12983_2018_287_MOESM5_ESM.pdf]
