# Supplementary material for: Delineating species along shifting shorelines: Tropheus (Teleostei, Cichlidae) from the southern subbasin of Lake Tanganyika
Source: Front Zool. 2018 Nov 13;15:42. doi: 10.1186/s12983-018-0287-4 (PMC6234679; doi:10.1186/s12983-018-0287-4)
Supplement: Supplementary file 4 — Output of cluster analysis performed in Structure. Groups are indicated as they occur along the shoreline with 1: T. sp. ‘maculatus’, 2: T. sp. ‘red’, 3: T. moorii ‘yellow’, 4: T. moorii ‘South’, 5: T. moorii ‘Southeast’, 6: T. brichardi ‘Kipili’, 7: T. sp. ‘Mpimbwe’. The northernmost specimen of T. sp. ‘red’ (Kikoti, loc. 4) is represented by the first sample of group 2, even though it appears within the distribution range of group 1. (PDF 317 kb) [file 12983_2018_287_MOESM4_ESM.pdf]

CLUMPAK main pipeline - Job 1536752824 summary

Major modes for the uploaded data:

K=1

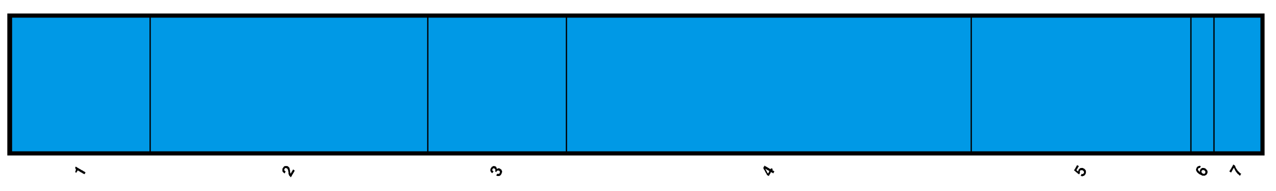

K=2

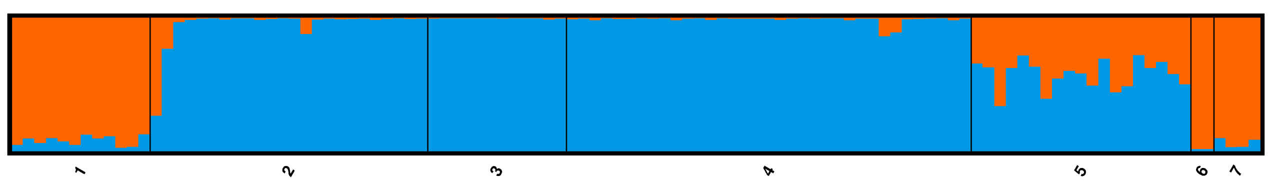

K=3

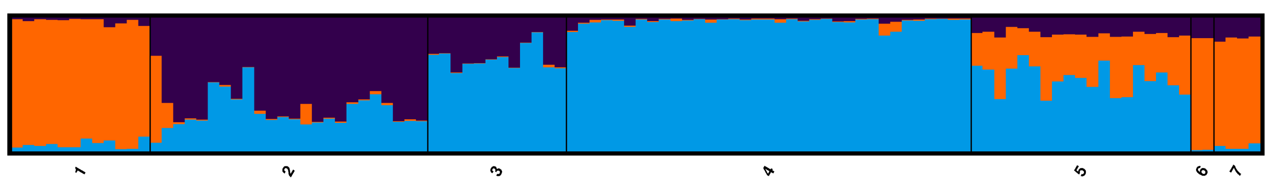

K=4

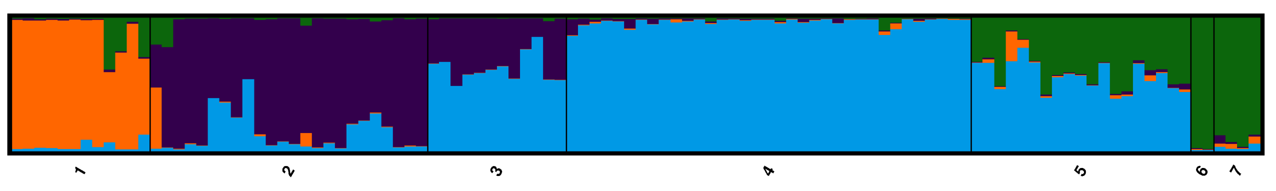

K=5

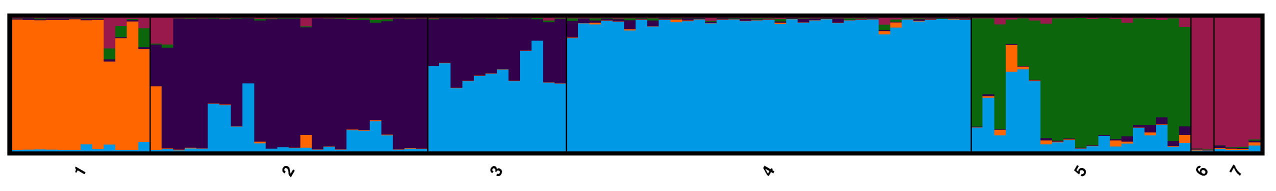

K=6

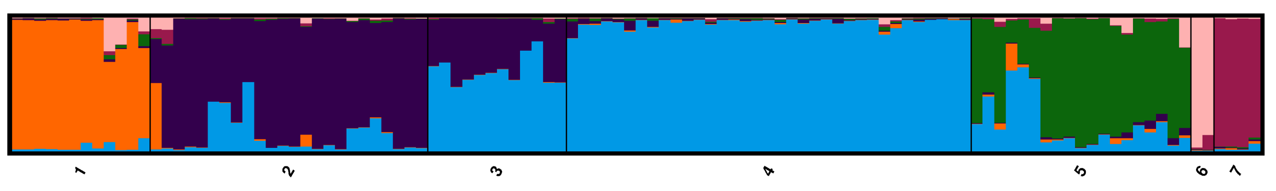

K=7

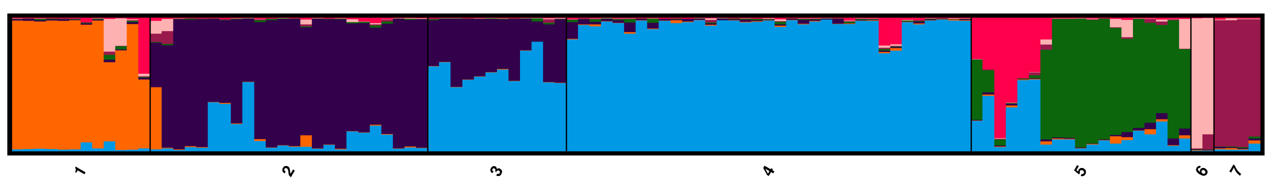

K=8

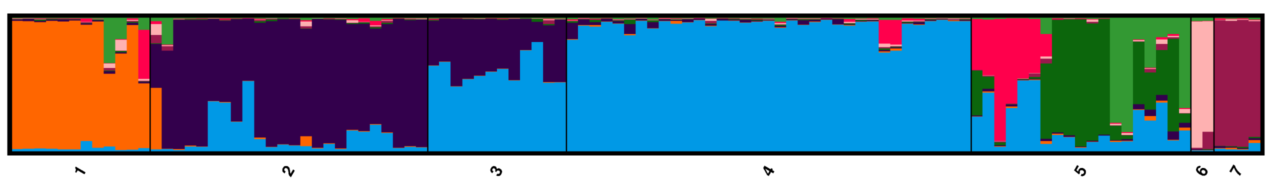

K=9

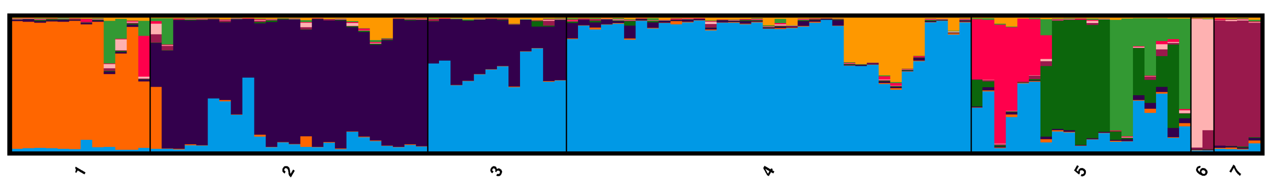

K=10

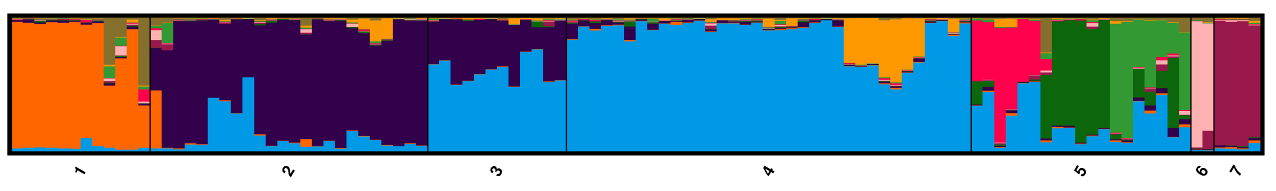

Minor modes for the uploaded data:

K=4 MinorCluster1

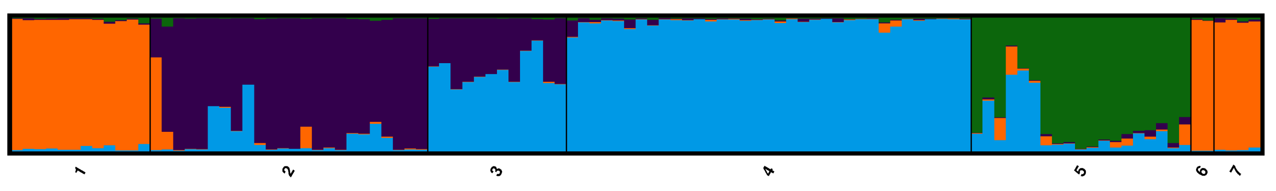

K=6 MinorCluster1

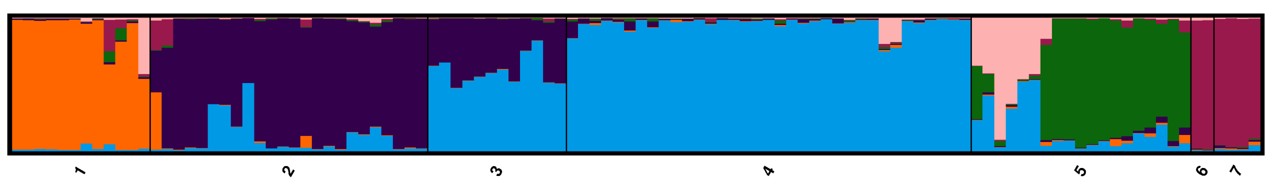

K=7 MinorCluster1

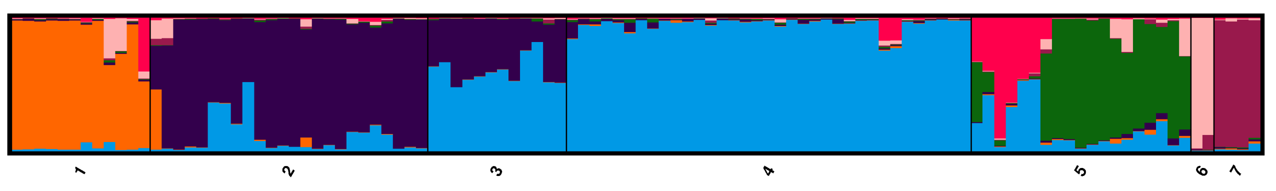

K=7 MinorCluster2

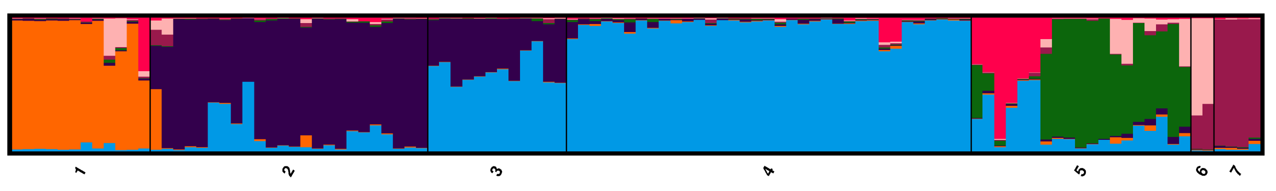

K=8 MinorCluster1

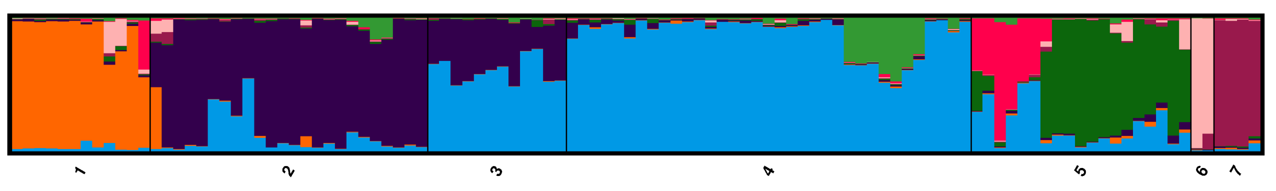

K=9 MinorCluster1

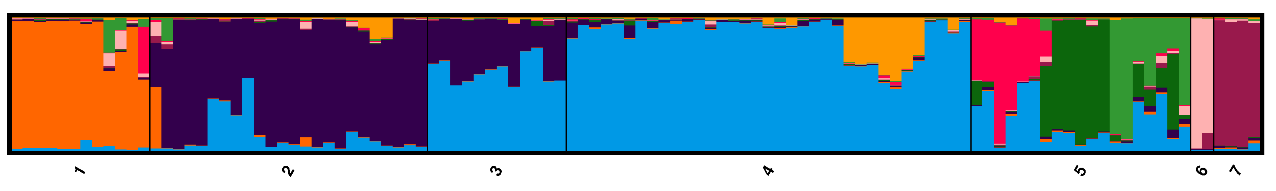

Division of runs by mode:

K=1	20/20
K=2	20/20
K=3	20/20
K=4	14/20, 6/20
K=5	20/20
K=6	14/20, 5/20
K=7	11/20, 6/20, 2/20
K=8	16/20, 3/20
K=9	17/20, 2/20
K=10	19/20
