# Supplementary material for: Delineating species along shifting shorelines: Tropheus (Teleostei, Cichlidae) from the southern subbasin of Lake Tanganyika
Source: Front Zool. 2018 Nov 13;15:42. doi: 10.1186/s12983-018-0287-4 (PMC6234679; doi:10.1186/s12983-018-0287-4)
Supplement: Supplementary file 6 — Morphological variation vs. geographical distance along the shoreline for the individual meristics and measurements. Measurements were expressed as percentages of head (for measurements taken on the head) or standard length, raw data were shown for meristics and for SL; the distance was taken along the shoreline (in km) starting from the northwesternmost locality. LOESS curves were calculated with a fixed smoothening parameter of 0.2. As this resulted in a straight line for ASp, 0.1 was used instead (*). (PDF 4487 kb) [file 12983_2018_287_MOESM6_ESM.pdf]

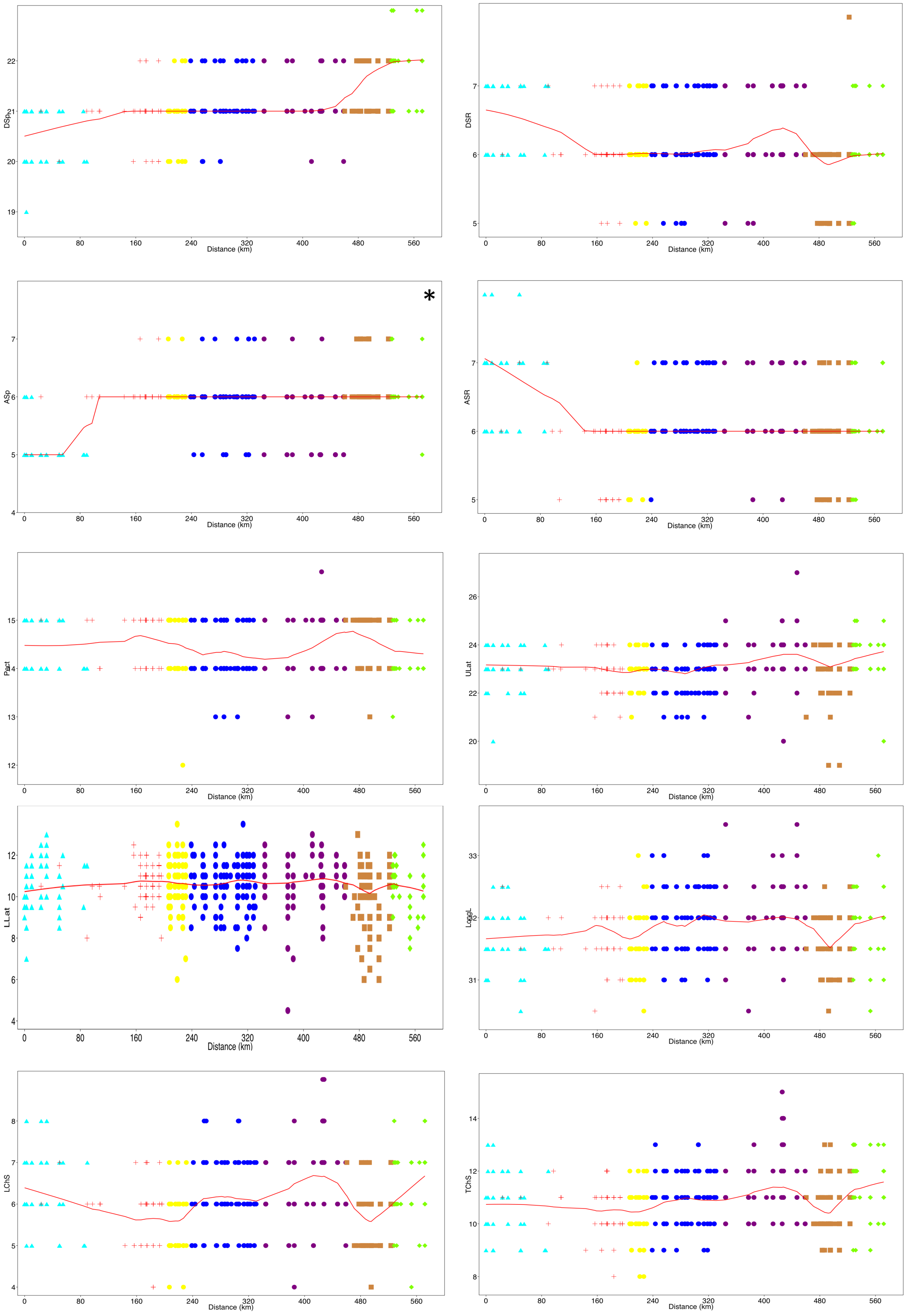

DSp
DSR
ASp
ASR
Pact
ULat
LLat
LoggL
LChS
TChS
Distance (km)
0 80 160 240 320 400 480 560
*

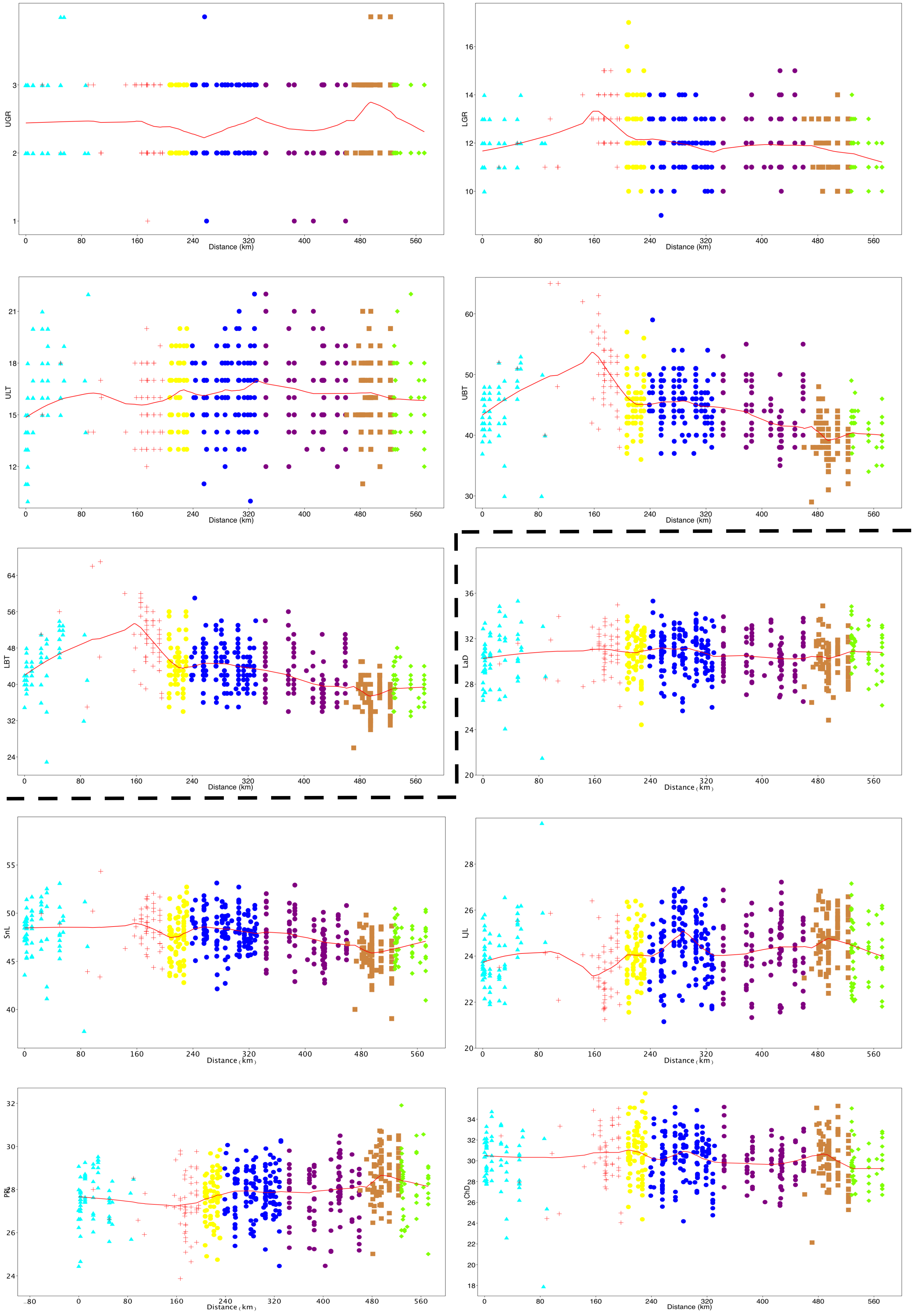
UGR
LGR
ULT
UBT
LBT
LaD
SnL
LJL
ChD
Distance (km)
0
80
160
240
320
400
480
560

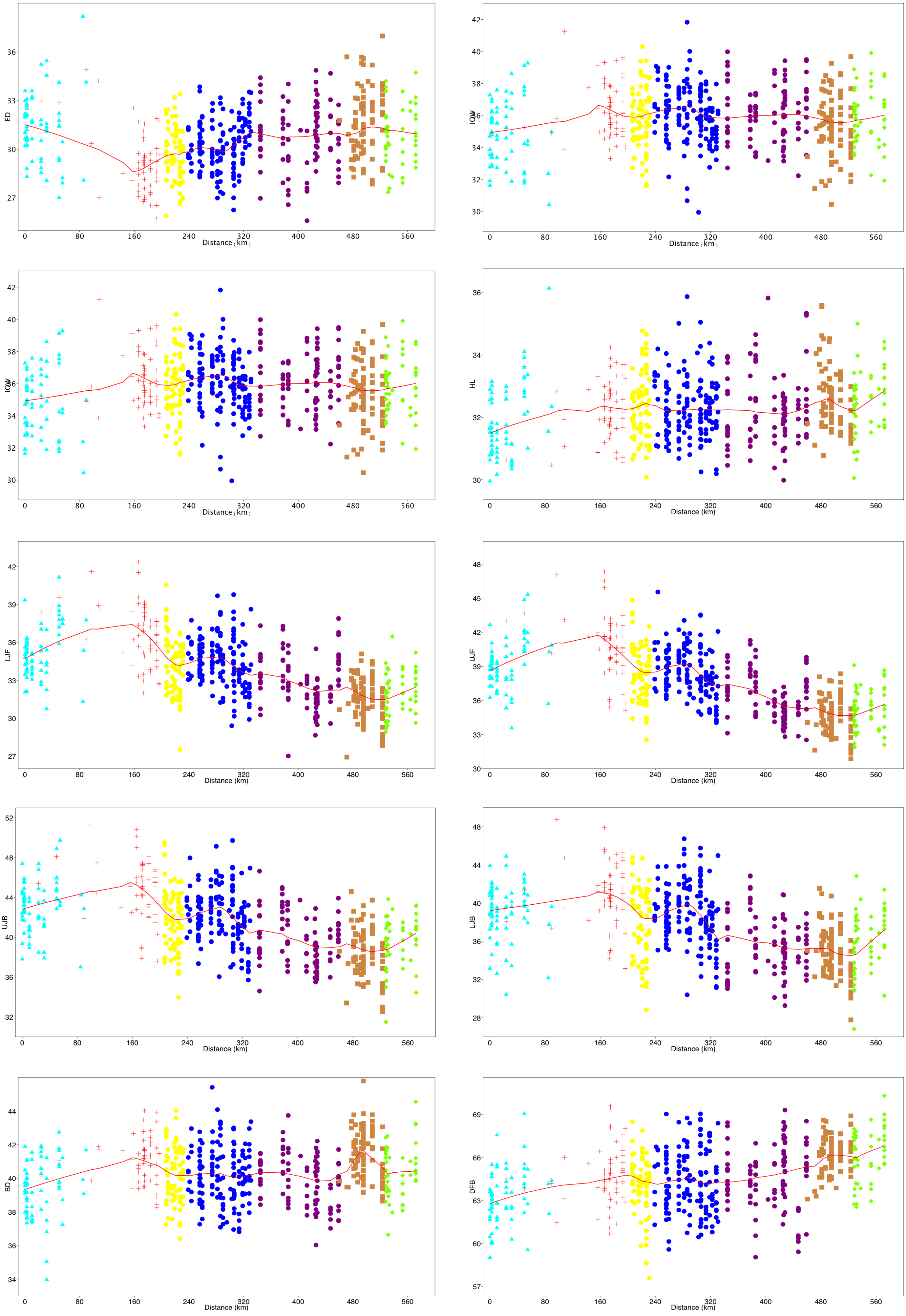

ED
IOW
IOW
HL
LJF
UJF
UJB
LJB
BD
DFB
Distance (km)

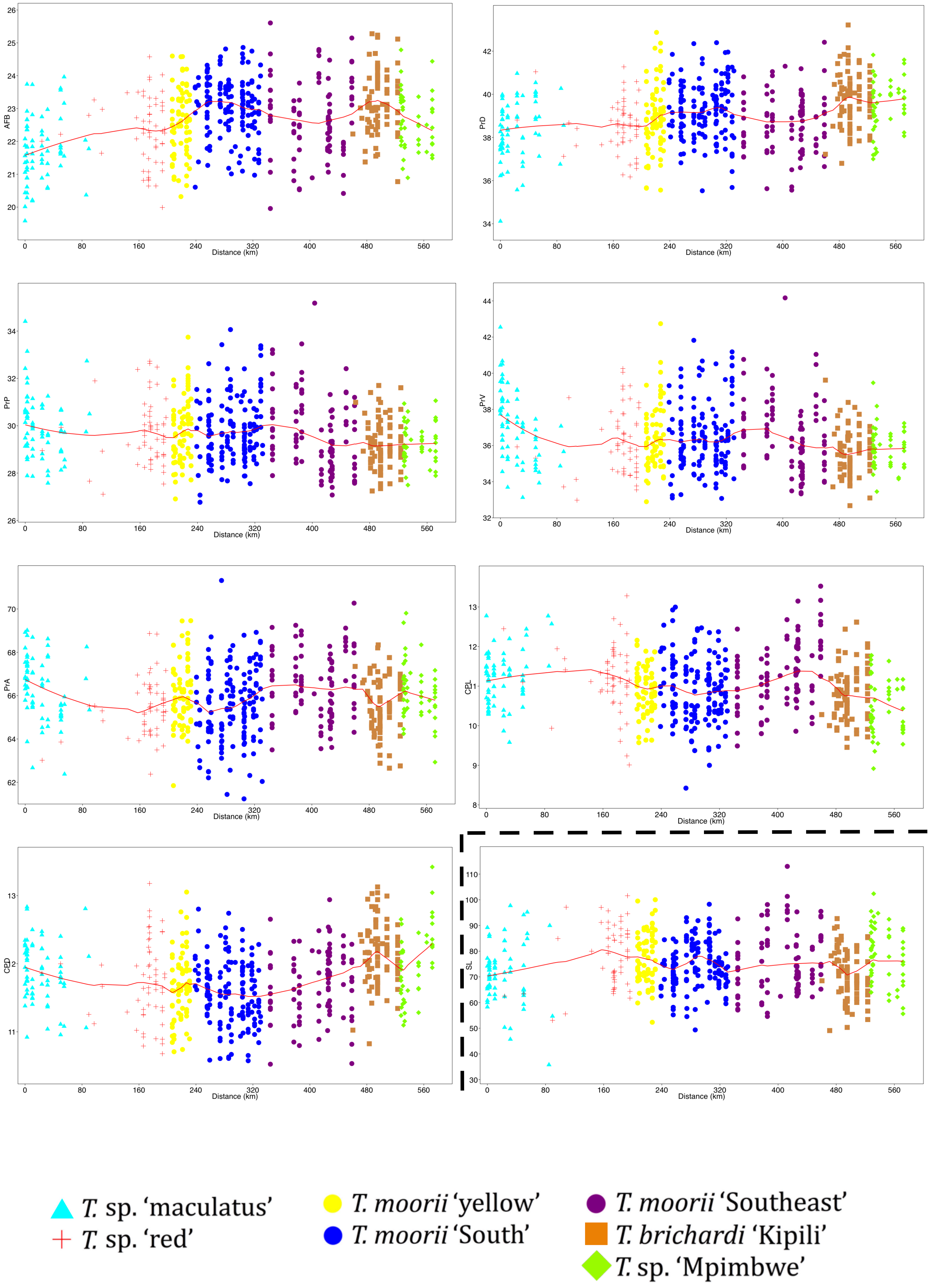
AFB
PrD
PrP
PrV
PrA
CPL
CPD
SL
Distance (km)
T. sp. 'maculatus'
T. sp. 'red'
T. moorii 'yellow'
T. moorii 'South'
T. moorii 'Southeast'
T. brichardi 'Kipili'
T. sp. 'Mpimbwe'
